# Particularities of allergy in the Tropics

**DOI:** 10.1186/s40413-016-0110-7

**Published:** 2016-06-27

**Authors:** Luis Caraballo, Josefina Zakzuk, Bee Wah Lee, Nathalie Acevedo, Jian Yi Soh, Mario Sánchez-Borges, Elham Hossny, Elizabeth García, Nelson Rosario, Ignacio Ansotegui, Leonardo Puerta, Jorge Sánchez, Victoria Cardona

**Affiliations:** Institute for Immunological Research, University of Cartagena, Cra. 5 # 7-77, Cartagena, Colombia; Khoo Teck Puat- National University Children’s Medical Institute, National University Health System, Singapore, Singapore; Department of Paediatrics, Yong Loo Lin School of Medicine, National University of Singapore, Singapore, Singapore; Department of Medicine Solna, Karolinska Institutet, Translational Immunology Unit, Stockholm, Sweden; Allergy and Clinical Immunology Department, Centro Médico- Docente La Trinidad and Clínica El Avila, Caracas, Venezuela; Pediatric Allergy and Immunology Unit, Children’s Hospital, Ain Shams University, Cairo, Egypt; Allergy Section, Fundación Santa Fe de Bogotá, Faculty of Medicine, Universidad de los Andes, Bogotá, Colombia; Federal University of Parana, Rua General Carneiro, Curitiba, Brazil; Department of Allergy and Immunology, Hospital Quirón Bizkaia, Bilbao, Spain; Department of Pediatrics, Graduate Program on Allergology, University of Antioquia, Medellín, Colombia; Allergy Section, Department of Internal Medicine, Hospital Vall d’Hebron, Barcelona, Spain

**Keywords:** The Tropics, Allergy, Asthma, Rhinitis, Atopic dermatitis, Helminthiases, Papular urticaria, Anaphylaxis, House dust mite, Natural history, Allergens

## Abstract

Allergic diseases are distributed worldwide and their risk factors and triggers vary according to geographical and socioeconomic conditions. Allergies are frequent in the Tropics but aspects of their prevalence, natural history, risk factors, sensitizers and triggers are not well defined and some are expected to be different from those in temperate zone countries. The aim of this review is to investigate if allergic diseases in the Tropics have particularities that deserve special attention for research and clinical practice. Such information will help to form a better understanding of the pathogenesis, diagnosis and management of allergic diseases in the Tropics. As expected, we found particularities in the Tropics that merit further study because they strongly affect the natural history of common allergic diseases; most of them related to climate conditions that favor permanent exposure to mite allergens, helminth infections and stinging insects. In addition, we detected several unmet needs in important areas which should be investigated and solved by collaborative efforts led by the emergent research groups on allergy from tropical countries.

**Electronic supplementary material:**

The online version of this article (doi:10.1186/s40413-016-0110-7) contains supplementary material, which is available to authorized users.

## Background

Allergy is an ecosystem determined disorder and variations in risk factors and triggers in different places is a common finding. These variations can be grouped into two major geographical environments that influence the evolution of allergic diseases: temperate zones and the Tropics. Nowadays most of theoretical foundations of allergy are derived from studies in temperate zones but important emerging information from the Tropics is showing that there are peculiarities in the natural history of allergies that deserve more systematic studies, not only for scientific reasons but also for designing strategies to improve the management and prevention of these disorders.

In general, the prevalence of allergic diseases in the Tropics is as high as is observed in temperate countries and in some regions even higher; however, food allergies and especially peanut allergy seem to be less common. It is essential to know the particular and shared risk and protective factors in order for the scientific community be able to stop the current allergy epidemic. Several risk factors for allergic diseases in the Tropics are similar to those in temperate zones but important exceptions justify particular analyses. One of the most striking epidemiologic differences is the perennial co-exposure to house dust mites (HDM) inhalation and helminth infections. The impact of this context on the inception, expression, evolution and management of most allergic diseases is increasingly understood but basic and clinical research is still needed to have a more accurate view of the natural history of allergic diseases in the Tropics.

As allergies that are highly dependent on environmental factors, it is expected that exposure to native (and sometimes exclusive) allergenic sources contribute to a high proportion of the differences. However, there are other less evident conditions that could modify allergy phenotypes in the Tropics. The effects of parasitic infections and the microbiota composition, which involve more complex mechanisms that potentially alter the allergic responses even to the conventional inducers, are examples of these conditions. In addition to specific tropical conditions, the urban centers of the Tropics share several risk factors such as air pollution and culturally-unique dietary patterns.

Because some of the current particularities in the Tropics are the result of limited hygiene conditions, it could be said that when future sanitary standards become the same as in the industrialized world, the differences would be determined basically by genetic factors, differences in microbiota due to natural climate and biodiversity, and currently unknown factors influencing the immune responses in the Tropics. In this review we present and analyze the results of a comprehensive search of the international and regional scientific databases, looking for those aspects of allergy in the Tropics that, based on scientific evidence, could be considered different from those observed in places with temperate climates. The literature search was conducted mainly in Pubmed and the search terms were selected according to the topics, for example, “asthma symptoms” and “allergen sensitization”. These were combined with “”tropical climate” or “tropics”, “Asia”, “Africa”, “South America”, etc. MeSH terms were also used and limited, for example, to “humans”, “English language”, etc. Most searches were accessed during 2015. The search results were then reviewed and selected based on basic, epidemiological or clinical relevance and studies done in the tropical and subtropical regions. The investigation was done to provide updated information to investigators and clinical practitioners and to evaluate the following hypotheses: a) Allergic diseases in the Tropics have particularities that deserve systematically designed studies and description in order to detect unmet research needs and provide better management of patients in the Tropics. b) Most of the particularities are related to climate conditions that favor the permanent exposure to mite allergens, helminth infections and stinging insects.

### The Tropics: particular climate and socio economic conditions influencing allergy

#### Climate and biodiversity

The Tropics (Torrid Zone) is one of the most dynamic and interesting regions of the world and contains the largest collection of living plants and animal species. The historical view that inhabitants from the temperate zone had about the Tropics has changed from not suitable for civilized habitation to being a paradise that harbors most of planet’s biodiversity which should be protected for the sake of humanity. For example, the Amazon rainforest covers most of the Amazon Basin of South America and comprises the largest and most biodiverse tract of tropical rainforest worldwide. In the past, most of the current tropical countries were colonies, mainly of European countries. During those times, the ancient view of the Tropics as a place of pestilences was reinforced by reports from physicians and scientists of particular (exotic) infectious diseases that were the foundations of “tropical medicine”. This concept is so strongly linked to infections that the study of other types of diseases, such as allergic diseases, had been delayed until several pernicious pre-conceived ideas were surpassed. Mean annual temperature in the Tropics is 28 °C and relative humidity 85 %. These conditions favor the existence of house dust mites and intestinal helminth infections, two of the most important environmental components of allergic diseases in the Tropics. In general, the temperate zone has moderate climate and four seasons but there are variations such as oceanic, Mediterranean and hemiboreal climates. In the Tropics there are also places of high altitudes where the climate is not typically hot and humid. These internal geographical variations in both zones could be the source of epidemiological differences, but in this review each zone will be taken as a whole.

#### Cultural beliefs and "folk medicine"

In tropical countries high rates of uncontrolled asthma and other allergies are frequently observed [1, 2], which is related to the particularities in perception and cultural attitudes towards disease, as well as education and socioeconomic conditions. The use of folk medicine (also known as traditional or indigenous medicine) to alleviate symptoms of asthma is well-accepted among different tropical cultures, but one concern about disease management with this type of medicine is that it reduces adherence to allopathic drugs that have been scientifically tested for allergic symptoms. While this theme has not been sufficiently addressed, there are many reasons to think that the poor use of the optimal treatment for allergic diseases has a socio-economical origin rather than a cultural preference of folk medicine by people living in the Tropics.

Studies about cultural beliefs regarding asthma in "Latino" communities show the co-existence of the biomedical model and ethnocultural beliefs to explain disease. Closeness to allopathic medicine is dependent on access to the health system, with the poorest being more attached to alternative options for treatment and health attention [3]. Qualitative research conducted in Latino communities also suggests that, in spite of using folk medicine products, parents or caregivers do not replace allopathic drugs for asthma treatment [4, 5]. In contrast, Bearison et al. found that, for Puerto Ricans, for example, reliance on home remedies for asthma is related to poor adherence with prescribed regimens [6].

#### Unawareness of disease and disease severity

An initial obstacle to accessing medication is the unawareness of being sick. Different studies in Latin America, for example, have found that most allergic conditions are frequently underdiagnosed [7]. In this sense, many people live with airflow limitation (and impaired qualify of life) but do not receive the appropriate treatment. Lack of information about allergic problems is expected in poor countries [8]. On the other hand, in different areas of the Tropics, patient perceptions of the severity of their own symptoms is far from reality [2]. In a recent multi-national survey in Latin America, Maspero et al. found that although 60 % of asthmatic patients reported their disease as well or completely controlled, a minority of them (8 %) met guideline criteria for well-controlled asthma [9]. The use of inhaled corticosteroids among patients with persistent asthma is scarce, which is related to the perception of disease as an acute condition only [10]. Another survey about asthma insights and attitudes, performed in 8 Asian-Pacific countries, found similar results to those in Latin America. Although the reasons are not well-defined it is possible that cultural beliefs and perceptions about disease origin may influence these findings. In Taiwan, for example, 76 % of surveyed patients admitted to having fear of inhaled steroids [11]. It is important to highlight that problems about perception of disease severity are not restricted to the Tropics and reinforcement on education about asthma control is a global need [12], but pertinent causes in each region must be identified.

#### Socio-economic conditions

There are 144 countries partially or fully located in Torrid Zone, comprising around 40 % of human beings. With the exception of Singapore and Hong Kong, most places are underdeveloped, with low or middle income economies where urbanism grows parallel to social inequalities. Urbanization is not always well planned but often guided by social problems in which rural inhabitants move to large urban centers. Although hygiene has improved in some cities, in general the poverty levels and low quality health policies impede the progress toward better hygiene conditions. In addition, in the bigger urban settings delinquency and violence induce behavioral changes among the population, especially the time spent indoors.

Poverty has a negative impact for asthma development and management [13, 14]. Elevated rates of uncontrolled asthma are found among the poorest [15]. Chronic asthma represents important out of pocket expenditures for families since access to health system is not universal, even though equality is the ultimate goal in health policies for most countries [16]. Moreover, control drugs for asthma are not easy to get in several countries. Beta-2 agonist inhalers are the “control” drug for many non-informed patients. Consequently, avoidable episodes of asthmatic crisis are frequent, and in turn, emergency room attendance rates are also high. Ultimately, the restrictions in expenditure for asthma management generates greater direct costs for the health system than those generated by providing free medication to uncontrolled patients or creating medical programs for asthma control and education. In countries where important changes in health policies about asthma management have been introduced, a dramatic reduction in asthma morbidity and mortality has been observed [17, 18]. High rates of uncontrolled asthma and other allergies and their underlying factors should be considered when classifying these problems during clinical and epidemiological surveys because they could be important confounding factors.

In summary, the climate, cultural and socioeconomic conditions in the Tropics facilitate an environment for the development of allergic diseases. Although socioeconomic conditions and particularly hygiene are modifiable risk factors, temperature and humidity levels are more constant and very appropriate for mite growth. The consequence of mite allergen exposure throughout the year is, probably the most important particularity of the Tropics with regards to allergy.

### The natural history of allergic disorders in the Tropics

The natural history of disease refers to its progression in an individual over time, in a course that involves stages of susceptibility, subclinical disease, clinical disease, and a final phase of recovery/remission, disability or death. Since the inception of allergy is largely dependent on the ecology and environmental effects, it is expected to find a broad diversity of predisposing and aggravating factors around the world. Studies in temperate areas often report that allergic diseases are more frequent in children with parental antecedents of atopy. Patients first develop cutaneous symptoms early in life, in the context of IgE sensitization to food allergens and aeroallergens, and then have respiratory symptoms in late childhood. They are also more frequent in children with early wheezing in the context of atopic sensitization. There is a male predominance that equilibrates gradually during childhood and cessation of symptoms is common but less likely if two allergic phenotypes co-exist (e.g. asthma and eczema) [19, 20].

Recent studies on patient phenotyping using biomarkers and clustering analyses have made it evident that there are many disease endotypes [21] and indeed more than one “natural history”, with environmental effects as major contributors and acting on a permissive genetic background. The problem of defining the natural history of allergy is not exclusive of the Tropics; in fact, the information available in temperate and industrialized countries is not enough to make accurate conclusions. Understanding the natural history will help to control the increasing trend of allergy prevalence, especially in tropical places, where asthma is also becoming a public health problem. As stated by The Global Asthma Report 2014: “The historical view of asthma being a disease of high-income countries no longer holds, most people affected are in low- and middle-income countries, and its prevalence is estimated to be increasing fastest in these countries” (http://www.globalasthmareport.org/burden/burden.php). Given the remarkable differences between tropical and temperate areas, certain environmental triggers and risk factors have particular relevance in the inception and progression of allergic diseases in the Tropics, as well as in defining the strategies for diagnosis and treatment [22].

#### Different phenotype paths

The tropical climate is among the particular factors that may influence the natural history of allergy. In this region of the world there are periods of the year with and without rain (dry season) [23] but not seasonal variations as described in temperate areas [24]. The high humidity is reflected in a high number of house dust mites, cockroaches, and molds and therefore a higher allergen load in homes and bedding materials. The rainy season is related to acute exacerbations of asthma by rapid changes to cold temperatures, the effects of thunderstorms on aerobiology or by promoting outbreaks of respiratory viral infections [25, 26]. Moreover, in regions with high temperatures, high humidity and rainfall through the year, the exposure to home dampness and molds in indoor bedrooms increases the risk for current symptoms of rhinoconjunctivitis [27]. Poor ventilation in indoor bedrooms is common, especially in deprived areas [28]. In some regions and because of the high temperature, windows and doors are open most of the time, and some studies revealed that in those settings natural ventilation is a protective factor for asthma [29]. The climate also predisposes to insect bites that induce papular urticaria.

As occurred in developed countries a few decades ago, urbanization seems to be critical in modifying allergic susceptibility, as suggested by the fact that prevalence of allergic diseases in urban and sub-urban areas of the Tropics are comparable to those found in affluent countries, but are very low or non-observed in rural communities [30–33]. Urbanization has a deep impact on the type of housing and bedding, access to green areas, diet, quality of water and air and exposure to pollutants. It should be pointed out that in many tropical areas urbanization often occurs in the context of poverty, and this combination promotes the exposure to noxious agents, fungal spores, and obsolete or low quality products that are regulated or even forbidden in the developed world, but modify allergy risk and promote reactions to nickel, cosmetics, detergents, rubber and pesticides in tropical settings [34, 35]. Wealth is also an important risk factor because even within short geographical distances the prevalence of IgE sensitization to aeroallergens is significantly higher in urban communities of high income compared to those economically deprived [36].

A remarkable finding is the high prevalence of recurrent wheezing in several tropical countries, compared to Europe and USA. Diverse viruses have been detected in children suffering acute respiratory illnesses in the Tropics [37]. Lower respiratory tract infections by respiratory syncytial virus cause 27 to 96 % of all acute wheezing hospitalizations in children under 6 month of age [38], and human rhinovirus is the most common pathogen in children with asthma and viral wheeze [39, 40]. In temperate areas, viral infections are also a frequent cause of wheezing but in tropical countries the admissions to hospitals are more frequent and the episodes more severe (http://www.globalasthmareport.org/burden/burden.php), [41]. In some tropical regions the viral-induced exacerbations of asthma are independent on the time of the year [42]. The oropharyngeal microbiome appeared to contain many more *Streptococci* in infants of rural Ecuador compared to Western Europe and the USA, and comparisons between healthy and wheezing children revealed significant differences in several bacterial phylotypes [43].

The few longitudinal studies analyzing the trajectories of allergic symptoms in the Tropics revealed particular aspects in the expression of allergic phenotypes. One remarkable finding is that the timeline in which IgE sensitization and symptoms evolve in the Tropics differs to the atopic march that has been described in some industrialized countries [44]. In the atopic march the symptoms often appear in a particular sequence starting with atopic dermatitis (AD) as the first manifestation of allergy in an infant, followed by food allergy, seasonal or perennial allergic rhinitis and finally asthma at late childhood [45]. Approximately 40-70 % of children with AD outgrow the disease by age 7 years but about half of them develop a respiratory allergy later in life [19]. In the Tropics, an observational study in the birth cohort FRAAT (Risk Factors for Asthma and Atopy in the Tropics), conducted in Cartagena (Colombia), revealed that none of children in the follow-up developed AD during the first two years of age, but 38 % of them have had wheezing and 15 % were recurrent wheezers [46]. Similar findings were obtained by a prospective study in Campinas (Brazil) in which 31 % of children at 12 months of age have had two or more wheezing episodes but there was only one case of AD [47]. The Ecuavida birth cohort in Esmeraldas (Ecuador) reported that 2.5 % of children by age 3 years have had recurrent episodes of eczema but 25.9 % have had wheezing and 7.1 % recurrent wheezing [48]. From those studies we can conclude that in some areas of the Tropics the dynamics of allergic manifestations is skewed to debut with respiratory symptoms. However, it is worth mentioning that birth cohorts in Malaysia [49] and Taiwan [50] have found that timelines for allergic symptoms in early childhood proceed according to the atopic march of temperate areas, suggesting that depending on the genetic background and the socioeconomic setting, the natural history can largely differ even within tropical regions. The concept of atopic march is controversial, and it has been recently described that only 7 % of children follow trajectories that resemble this pattern [51]. Since very few longitudinal studies have prospectively followed the evolution of allergic phenotypes in the same individuals, most conclusions on the natural history have been derived from cross-sectional studies. More studies considering careful phenotype assessment, bias in sample selection and heterogeneous exposure to infectious agents are highly needed. The following points could summarize the particularities in the natural history of allergic conditions in the Tropics. All of them will be further analyzed in each section of this review.

- Early respiratory symptoms are more frequent than AD in children. The reason is still unclear but perennial mite exposure, early helminthic infections and genetic factors may play a role.
- In general, there is a low prevalence of physician-diagnosed AD, varying from infrequent or non-observed in some places to common in others. Remarkable differences in AD prevalence can be observed even in the same country.
- Allergic skin reactions do occur in the Tropics and in general are the same as in temperate countries, but in some regions they show remarkable differences regarding to clinical presentation and risk factors [52]. Parasite migration or treatment with antiparasitic drugs can induce urticaria.
- Papular urticaria by insect bites is more common than in the rest of the world.
- Early exposures to geohelminths infections modify the expression of allergic diseases, including their frequency and severity [53, 54].
- The symptoms of rhinoconjunctivitis reported in urban centers of developing countries are more severe than those reported in developed countries [55].
- IgE sensitization to aeroallergens occurs early in life and at higher frequencies compared to temperate areas [13, 56, 45].
- The main sensitizers in allergic patients living in tropical urban environments are HDM [57] and cockroach [58–60]. IgE response in airway allergy is dominated by a single class of allergen source, dust mites.
- There are no significant differences in the allergen levels during the year [61].
- The frequency of IgE sensitization and the strength of IgE levels to cross-reactive pan-allergens like tropomyosin are higher compared to temperate areas [62].
- Sensitization to pollens is less frequent and less intense than to mite allergens. In regions with transition to subtropical areas the role of pollens is important, although the pattern of sensitization is different to that observed in Europe [63, 64].
- IgE sensitization to food allergens is frequently detected without symptoms and aeroallergen sensitization is not usually preceded by food sensitization. The sources of food allergens are different; for instance hypersensitivity to shellfish, fish and fruits is more common than reactions to nuts, peanut and wheat [65–67].

#### Early respiratory symptoms and allergen sensitization in the Tropics

It is well recognized that wheezing is frequent in the Tropics and several factors have been implicated including viral infections, elevated endotoxin levels and pollutants [46, 68, 69]. However, there are few studies on the atopic component of early wheezing in the Tropics, which has made the analysis of this important point based on reports from temperate countries. A birth cohort conducted in Salvador-Bahía (Brazil) with full evaluation by physicians and skin prick tests reported that 25 % of children by age 5 years have allergic respiratory symptoms [70]. In addition, in a nested case-control study in children from Esmeraldas (Ecuador) it was found that 32.69 % of wheezers and 10.8 % of controls living in urban zones were HDM sensitized as detected by serum specific IgE, a highly significant difference [71]. Furthermore, the evaluation of early sensitization in the FRAAT cohort (Cartagena, Colombia) revealed that 33.3 % of 3 years old children were sensitized to *B. tropicalis*, and this was a risk factor for wheezing [13]. IgE sensitization to *Ascaris* has also been associated with wheezing in children sensitized to mites or food allergens [71, 72]. The influence of allergen sensitization on wheezing has been detected, although at a low rate, even in parasited communities and using skin testing for atopy diagnosis [73]. All of these finding suggest the great importance of early sensitization to mite and *Ascaris* allergens, although a proportion of wheezing is driven by non-IgE pathways, more likely viral infections. However, as will be discussed later (see Allergen sensitization and asthma symptoms) sensitization rates increase with age and in older children reach impressive levels. In addition, maternal antecedent of allergic disease is an independent risk factor for wheezing and asthma in the offspring [74]. It has been also found that maternal effects are significant for boys but not for girls [75].

In summary, there are few studies addressed to establish the natural history of allergic disorders in the Tropics. Although not well investigated, childhood viral infections seem to be as prevalent as in temperate zones, being an important cause of wheezing, the commonest respiratory symptom in infancy. In addition, there are reports suggesting that it is associated with IgE sensitization to mite and helminth allergens. One striking finding has been the low prevalence of doctor diagnosed AD in several populations and the absence of the “atopic march” described in some temperate countries. The progress and severity of asthma is influenced by helminth infections. It is more frequent in urban areas where helminths are less prevalent and with lower parasitic load.

### The prevalence and particularities of allergic diseases in the Tropics

Because of the increasing trends of allergy in industrialized countries, one frequently discussed issue regarding allergy in the Tropics is its prevalence. Although in the past it was difficult to understand, now it is generally accepted that allergic disorders are very common in underdeveloped countries of the Tropics. In addition to lack of information, there were several reasons for this belief. The first was the “tropical diseases” idea: in this zone the diseases were limited mainly to infections. This was reinforced by the influence of the hygiene hypothesis, so well accepted that for many minds it ceased to be a hypothesis and made it difficult to reconcile the existence of allergic problems with the high prevalence of bacterial and viral infections in the same regions. Another reason was the increasing number of basic science reports showing the important immunomodulatory effects of helminth infections, which has been wrongly generalized to conclude that allergies are rare in places where helminthiases exist.

Interestingly, during the last years several well conducted epidemiological surveys have demonstrated the high prevalence of allergic diseases in tropical underdeveloped countries. In addition, the hygiene hypothesis has failed to resolve important contradictions with empirical data and finally, the immunostimulatory effects of helminthiases have been evaluated at the population level. Based on the limited historic information available, it is very difficult to define if there is an allergy epidemic in the Tropics or how long these diseases were present. However, since tropical centers started to be involved in the Study of Asthma and Allergies in Childhood (ISAAC), it was evident that asthma and rhinitis were very frequent in some cities. We do not know if this trend came from long or even if the natural exposure to mite allergy induced underdiagnosed allergy symptoms. In the following five sections we will review the prevalence and relevant clinical aspects of some allergic conditions. Asthma and rhinitis as well as atopic dermatitis will be analyzed by including both ISAAC and regional studies from the Tropics.

### Asthma and rhinitis in children

Asthma and rhinitis are among the most common chronic illnesses of childhood, with significant impaired quality of life [76–80]. Studies worldwide have shown an increasing trend in their prevalence over the last decades. ISAAC Phase I (conducted in the early 1990s’) and Phase III (2001–2003) detected an overall increase in the prevalence of rhinoconjunctivitis which was more marked in older children [81]. There was an increase in asthma prevalence as well, especially in regions where it was lower [82]. However, there are regional surveys, some relatively recent, that may be able to shed more light on other local factors pertaining to allergic diseases. In fact, interesting information resulted when analyzing the pattern of asthma [83] and rhinoconjunctivitis [81] within the tropical ISAAC centers; comparing these patterns with the subtropical and temperate ISAAC centers and reviewing the regional surveys in the Tropics pertaining to the patterns of asthma and rhinoconjunctivitis (Table 1, Additional file 1: Table S1 and Additional file 2: Table S2).

**Table 1:** Overview of children studied in tropical centers in ISAAC Phase III

Outcome	Age 13–14 years			Age 6–7 years		
	Number of centers	Mean prevalence, %	Standard deviation	Number of centers	Mean prevalence, %	Standard deviation
Current rhinitis	86	32.6	12.5	49	24.1	11.2
Change per year in Current Rhinitis	26	+0.26	1.15	17	+0.26	0.84
Hay Fever ever	80	20.8	14.6	46	15.7	10.1
Change per year in Hay Fever ever	22	+0.95	1.72	15	+0.73	1.46
Current rhinoconjunctivitis	86	15.2	6.9	48	9.5	5.3
Change per year in Current rhinoconjunctivitis	26	+0.06	0.99	17	+0.19	0.29
Current wheeze	86	13.0	6.8	49	12.8	8.5
Asthma ever	86	13.0	7.1	49	10.9	8.4

#### Comparison of tropical, subtropical and temperate ISAAC centers

Prevalence of current rhinitis and current rhinoconjunctivitis between Phase I and Phase III increased in the Tropics and subtropics, and was higher in subtropical centers compared to tropical centers, for all children as well as children aged 13–14 years, but not in children aged 6–7 years. In contrast, the diagnosis of asthma ever was lower in the subtropics compared to the Tropics (Table 2). Comparing the Tropics and temperate centers, the symptoms of current rhinitis, current rhinoconjunctivitis, and a diagnosis of hay fever ever were higher in the Tropics, especially in children aged 6–7 years. Symptoms of current rhinoconjunctivitis and change in prevalence of hay fever ever were significantly higher in the Tropics compared to the temperate centers, in children aged 13–14 years. When comparing subtropical with temperate centers, similar results were obtained.

**Table 2:** Significant differences between tropical, subtropical and temperate ISAAC centers

Outcome	Mean (SD), tropics	Mean (SD), subtropics	Mean (SD), temperate	*p*-value (difference between all groups)	Mean difference (tropics vs subtropics)	*p*-value (tropics vs subtropics)	Mean difference (tropics vs temperate)	*p*-value (tropics vs temperate)	Mean difference (subtropics vs temperate)	*p*-value (subtropics vs temperate)
Current rhinitis: Whole Cohort	29.5 (12.7)	28.5 (11.8)	25.0 (9.1)	0.001	1.03	NS	4.47	0.001	3.44	0.028
Current rhinitis: Age 13–14 years	32.6 (12.5)	32.5 (12.1)	29.4 (8.5)	NS	0.01	NS	3.12	NS	3.11	NS
Current rhinitis: Age 6–7 years	24.1 (11.2)	21.8 (7.9)	18.5 (5.4)	0.002	2.31	NS	5.58	<0.001	3.28	NS
Change per year in Current rhinitis: Whole Cohort	0.26 (1.03)	0.93 (1.30)	0.32 (0.93)	0.02	−0.67	0.01	−0.06	NS	0.61	0.009
Change per year in Current rhinitis: Age 13–14 years	0.26 (1.15)	1.07 (1.46)	0.29 (1.11)	0.046	−0.81	0.03	−0.04	NS	0.78	0.018
Change per year in Current rhinitis: Age 6–7 years	0.26 (0.84)	0.59 (0.77)	0.36 (0.56)	NS	−0.33	NS	−0.10	NS	0.23	NS
Hay Fever Ever: Whole Cohort	18.9 (13.3)	21.2 (15.2)	14.6 (10.7)	<0.001	−2.14	NS	4.37	0.004	6.51	<0.001
Hay Fever Ever: Age 13–14 years	20.8 (14.6)	24.2 (14.5)	18.4 (11.7)	NS	−3.39	NS	2.44	NS	5.83	0.017
Hay Fever Ever: Age 6–7 years	15.7 (10.1)	16.0 (15.2)	9.0 (5.5)	<0.001	−0.31	NS	6.70	<0.001	7.01	0.002
Change per year in Hay Fever Ever: Whole Cohort	0.86 (1.60)	0.69 (1.05)	0.37 (0.61)	0.025	0.18	NS	0.49	0.01	0.31	NS
Change per year in Hay Fever Ever: Age 13–14 years	0.95 (1.72)	0.76 (1.17)	0.38 (0.70)	NS	−0.19	NS	0.58	0.033	−0.38	NS
Change per year in Hay Fever Ever: Age 6–7 years	0.73 (1.46)	0.47 (0.62)	0.36 (0.47)	NS	0.25	NS	0.37	NS	0.11	NS
Current rhinoconjunctivitis: Whole Cohort	13.1 (6.9)	13.3 (7.4)	10.7 (5.3)	0.001	−0.13	NS	2.43	0.001	0.26	0.005
Current rhinoconjunctivitis: Age 13–14 years	15.2 (6.9)	16.1 (7.3)	13.2 (5.0)	0.015	−0.95	NS	2.00	0.031	2.95	0.01
Current rhinoconjunctivitis: Age 6–7 years	9.5 (5.3)	8.6 (5.0)	7.0 (3.0)	0.011	0.87	NS	2.42	0.003	1.55	NS
Change per year in Current rhinoconjunctivitis: Whole Cohort	0.11 (0.79)	0.59 (0.77)	0.12 (0.57)	0.006	−0.48	0.005	−0.01	NS	0.47	0.002
Change per year in Current rhinoconjunctivitis: Age 13–14 years	0.06 (0.99)	0.66 (0.86)	0.09 (0.71)	0.027	−0.61	0.018	−0.03	NS	0.58	0.011
Change per year in Current rhinoconjunctivitis: Age 6–7 years	0.19 (0.29)	0.41 (0.52)	0.17 (0.26)	NS	−0.22	NS	0.02	NS	0.24	NS
Current wheeze: Whole cohort	12.9 (7.5)	11.6 (6.5)	12.7 (6.5)	NS	1.32	NS	0.85	NS	−0.48	NS
Current wheeze: Age 13–14 years	13.0 (6.8)	11.5 (5.9)	12.9 (7.2)	NS	1.53	NS	0.05	NS	−1.48	NS
Current wheeze: Age 6–7 years	12.8 (8.5)	11.9 (7.6)	10.8 (4.9)	NS	0.97	NS	2.02	NS	1.05	NS
Asthma Ever: Whole Cohort	12.2 (7.7)	8.9 (5.0)	11.2 (8.3)	0.009	3.36	0.002	1.04	NS	−2.32	0.028
Asthma Ever: Age 13–14 years	13.0 (7.1)	9.8 (5.1)	12.3 (8.6)	NS	3.23	0.019	0.70	NS	−2.53	NS
Asthma Ever: Age 6–7 years	10.9 (8.4)	7.4 (4.5)	9.6 (7.5)	NS	3.54	0.048	1.33	NS	−2.21	NS

#### Regional, non-ISAAC studies on asthma and rhinitis

A total of 13 surveys [28, 84–97], all cross-sectional and describing asthma or rhinitis in children in their respective communities within tropical countries was found (Additional file 2: Table S2). Twelve reported the prevalence of asthma and/or wheeze in the last 12 months and two presented the prevalence of rhinoconjunctivitis. With one exception [84] all recruited at least a thousand or more children. Four studies looked at children in rural communities [84, 87, 90, 95]; the rest were in urban environments. Most of these surveys employed ISAAC questionnaires and definitions in their methodology. Three used different criteria to define asthma. Response rates were excellent in all except one study, where only 48 % of subjects returned the questionnaires [92].

As in ISAAC surveys, the rates of current wheeze and/or asthma varied widely, ranging from 3 % in Tumbes [87] to around 27 % in Costa Rica [28]. In the four rural studies, asthma prevalence was generally low, ranging from 3 to 10.5 %, with the exception of the study involving the Warao Amerindians (26 %) in Venezuela [84]. Prevalence of asthma, symptoms of rhinitis and rhinoconjunctivitis varied widely. In two large studies in Mexico [85, 86], asthma was most prevalent in Villahermosa (10.2 %), rhinitis and rhinoconjunctivitis were highest in the Southeast Federal District of Mexico (53 % rhinitis, 25.7 % rhinoconjunctivitis) and lowest in Toluca (asthma 5.9 %, rhinitis 18.6 %, rhinoconjunctivitis 7.3 %).

The ISAAC studies encompassed children all over the world, and Mallol et al. [98] detected a weak but significant inverse relationship between latitude and prevalence of asthma and rhinoconjunctivitis. The available data from the Tropics suggest that for current rhinoconjunctivitis, current wheeze, hay fever-ever and asthma-ever, the effect of latitude is important, especially in children aged 13–14 years. This implies that the closer to the Equator, the higher the prevalence of symptoms in children as they get older. These findings could be associated to particular conditions observed in the Tropics.

One is the effect of ultraviolet-B radiation, which is linked to Vitamin D and is assumed to be associated with latitude but remains a controversial issue. The Tasmanian Longitudinal Health Study [99] found that proximity to the equator and higher ultraviolet-B exposure was associated with higher rates of atopy and atopic sensitization. In contrast other authors [100] did not find association between Vitamin D, ultraviolet radiation and asthma. Furthermore, a lower serum level of 25-hydroxy vitamin D has been associated with higher risk of asthma and more severe asthma in both the Tropics [101, 102] and other countries [103], which contradict the assumptions of the relationship between latitude and vitamin D status. Finally, latitude might have an indirect effect on the development of atopic symptoms through alteration of the magnitude [104] of protective or risk factors for disease; therefore, further research in this area is mandatory.

Another, more documented factor is the effect of house dust mites, which are the major aeroallergens in the Tropics in terms of prevalence and contribution to atopic disease, as has been demonstrated in multiple studies [60, 105–115]. House dust mite sensitization also appears to increase with age [105], again suggesting a time-dependent risk of sensitization. Though the dual allergen hypothesis [116] was initially formulated to explain peanut allergy, it might also hold true for dust mite allergy: children (and adults) in the Tropics would have considerable cutaneous (as well as possible inhalation) exposure to dust mite allergens when they sleep. This risk of sensitization would be exposure-dependent and therefore increases with age, which would explain the observations of Chiang et al. [105] as well as the effect we found in older children in the Tropics.

The differences between temperate regions and the tropic/subtropics are quite marked. It is also noteworthy that between Phase I and Phase III, the increase in prevalence of current rhinitis and rhinoconjunctivitis was highest in the subtropics compared to either Tropics or temperate regions. Such differences highlight two key points. One is the possibility that other factors beyond sensitization to dust mites account for the rapid rise in atopic symptoms in the subtropics, which merits further study on the pathogenesis for atopic disease and its relationship with the environment in this region alone. Given that environmental factors influence the success of any intervention intended to change the trends of atopic disease, current data from the general scientific literature (almost all from temperate climates) may not be applicable to children in the Tropics. Further study in this part of the world is needed especially in the areas of environmental influences and environmental interventions.

In summary, asthma and rhinitis prevalence in children living in the Tropics is higher than that reported in temperate countries for the same age groups. An overall increase of allergies from ISAAC I to III was observed, similar to that in temperate countries. In addition a trend for increasing allergy symptoms in places closer to the Equator was observed, suggesting an effect of latitude that deserves more study.

### Atopic dermatitis

AD is widely distributed, particularly among children under 5 years. There is growing information about increase of AD prevalence, nevertheless high variation in the frequencies is observed [117]. The most known international effort evaluating the epidemiology of AD is the ISAAC study, using a questionnaire completed by the participants based in the Williams diagnostic criteria [118]. Several centers from tropical countries participated in ISAAC phase I and III, and for some the prevalence of AD was found higher than in other regions, with a mode of 15 %. This suggests that genetic and environmental factors have an important effect in the development of AD. Since the surveys were completed by the participants other explanations like selection bias and misidentification of the disease by the patient should be considered. In this section we compare the prevalence of AD in tropical countries as reported by the ISAAC and surveys using other commonly diagnostic tools. In addition, we evaluate the impact of infectious skin problems on the differential diagnosis of AD.

#### Challenges for the diagnosis of AD in the Tropics

For diagnosis, the presence of pruritus and at least two of the other criteria is essential. However, these symptoms are not pathognomonic of AD and could be present in other skin diseases. In tropical countries, especially in underdeveloped areas, other causes of pruritus and lichenified injuries are common (scabies, papular urticaria, seborrheic dermatitis, miliaria, infection-induced rashes, *etc.*). Miliaria, also called “sweat rash” or “heat rash”, is a skin disease marked by small and itchy rashes [119], common in hot and humid conditions, such as in the Tropics and during the summer in subtropical regions. Although it affects people of all ages, it is especially common in children and infants. Papular urticaria is caused by hypersensitivity reaction to insect bites. Scabies is a common skin disease especially in children under 10 years (9 %) caused by infection with the mite *Sarcoptes scabiei* [120, 121]. The characteristic symptoms of a scabies infection include intense itching and superficial burrows.

In South India, Brazil and Turkey, more than 30 % of children under 7 years had miliaria and the main differential diagnosis was AD [122–124]. These studies showed that the etiologies of around 50 % of dermatoses were infections such as scabiosis; nevertheless between 50 and 80 % of cases were initially diagnosed as AD, which was the second most frequent skin problem with prevalence between 6.5 and 12 %. It is also common that some of these diseases appear simultaneously, hindering the diagnosis and appropriate treatment. In Nigeria one study including 1.066 children under 12 years observed that about 20 % of children with dermatoses have two or more skin disease [125]. AD was virtually absent (<1 %) but 60 % of patients had another skin disorder with scabiosis being among the most common.

#### ISAAC results on AD

There were seven questions of the ISAAC survey focused on AD; in addition two combinations of symptoms were evaluated: current symptoms and current symptoms of severe AD. Five of the seven questions were focused on pruritus, and positive response could suggest that the child has or had dermatitis. However excepting the question about lesions distribution none could differentiate if the itching was secondary to other common skin conditions, especially those described above. According to ISAAC Phase I and Phase III results, the prevalence of “eczema ever” in tropical countries among children aged 6–7 years, varied from 5 % in Jodhpur (India) to 44 % in Quito (Ecuador). The presence of “current eczema” varied from 0.9 % in Jodhpur to 22.5 % in Quito. In children between 13 and 14 years, the prevalence ranged from 0.2 % in Tibet (China) to 24.6 % in Barranquilla (Colombia). All centers in tropical areas from Latin America and Asia showed a significantly higher risk of current symptoms. La Habana (Cuba) and San Pedro Sula (Honduras) were the centers with the highest risk of current symptoms of severe eczema in tropical regions (two out of three patients). In summary, the ISAAC studies provide information about the prevalence of AD in the general population in several countries to compare the results and identify possible risk factors. However, the age of the patients who underwent the questionnaire and the type of questions answered by self-reporting may lead recall bias especially in tropical regions, where many other itchy skin diseases are highly prevalent.

#### Comparison of ISAAC and other epidemiological studies in tropical countries

In tropical areas, one of the most highlighted results of ISAAC Phase III was the increasing prevalence of AD compared with Phase I, especially in Latin American countries. A higher frequency of current dermatitis among children 6 to 7 years (24 %) was observed in Barranquilla-Colombia. These results contrast with data from a similar study done in this city by Dennis et al., the same year with the same ISAAC questionnaire and similar size population where a parent-reported prevalence of current eczema was less than 7 % [7]. In that study, the prevalence of medical diagnosed AD was less than 2 %. The frequency of severe eczema was similar between studies. Ten years later, in a second study Dennis et al. using the same methodology found that the prevalence of parent-reported AD in Barranquilla was 11 % and medical diagnosis less than 7 % [126].

In Cartagena, data from the FRAAT birth cohort showed that none of the children at age of 3 years had developed AD [46]. Given that the ISAAC study carried out the survey among families with children over 6 years and FRAAT results were in a population with less than 3 years, these results suggest that in some cities in Latin America the onset of dermatitis is later in life (>3 years) similar to that found in some tropical countries located in the South-East of Europe [127]. However, the follow-up study FRAAT at 5 years has shown no change in the prevalence of dermatitis. Another possible explanation may be the methodology of data collection; in the FRAAT cohort, ISAAC questionnaire was applied together with a physician examination.

In a study from Cuba, Gruchalla et al. showed marked differences between results of ISAAC questionnaire or physician diagnosis of dermatitis [128]. A total of 398 children from five randomly selected primary schools were included in the study; age range was 5–13 years (median 8 years). Parents or guardians were interviewed by using an extended version of the ISAAC questionnaire. Additionally, a physician made a physical examination of each child. A thorough inspection of the skin for signs of AD was done. For AD, the answers to the seven core questions yielded symptom percentages between 8 and 26 %. Using the ISAAC definition for AD [118] 9 % of the children were considered as suffering from AD, and 3.5 % as severe AD. When physician evaluation was done, only five children (<2 %) showed clinical signs of AD, which means a significant difference in prevalence as reported by ISAAC questionnaires. In addition, by applying the score of Yamada et al. [129] 19 % had AD. These data show a significant variation in prevalence according to the questionnaire used to define AD and in both systems dermatitis prevalence was overestimated as compared to the clinical examination.

In summary, although there are several population studies on AD, very little is known about the actual prevalence, especially outside Europe. The ISAAC permits comparisons to estimate the global burden of AD, to generate new hypotheses on disease causation that may only become apparent when prevalence data on a global scale are examined, and to test existing hypotheses regarding disease etiology on a wider scale. Nevertheless, ISAAC has limitations and the results have to be taken with precaution especially in tropical regions where other skin itchy disorders are frequent. Studies that include objective skin examinations are required to confirm the prevalence in these regions.

### Urticaria

Urticaria is a highly prevalent condition with various clinical manifestations [130–132]. When the symptoms are present for less than six weeks it is considered acute urticaria (AU), otherwise it is termed chronic urticaria (CU) [131, 133]. Although accurate data on the prevalence of urticaria is unavailable, it ranges from 0.03 to 11.3 % [130, 134]. It may affect between 15 and 30 % of individuals at some point of their life [133, 135–138]. Around 25 % of AU can evolve to CU forms [138]. CU resolves spontaneously in 30–55 % of patients within 5 years [139]. Autoimmune disturbances are present in 40 to 45 % of patients with CU. Recently urticaria management guidelines have been published in both temperate and tropical countries, wherein definition, classification, risk factors and treatment are extensively described [130, 138, 140].

Sánchez-Borges et al. investigated the demographic and clinical characteristics of urticaria patients attending allergy clinics in Caracas, Venezuela during a three-year period; patients with urticaria were 21.8 % of all consulting to the allergy services, 40.1 % AU and 59.8 % CU. AU was more frequent in adult female subjects; 31 % presented generalized urticaria. Papular urticaria occurred more frequently in children (39.4 %) than in adults (9.9 %), whereas drug-induced urticaria were observed mainly in adults [136]. In Mexico City, Cariño et al. performed a retrospective, analytical, descriptive study of patients treated at the Department of Allergy and Clinical Immunology. They included 1913 patients, of whom 186 (9.7 %) were diagnosed as urticaria, angioedema or both, 47 were men (25.2 %) and 139 women (74.7 %). The highest prevalence was found in adults (90.3 %), the mean age was 37.4 years [141]. The results of these studies are similar to those reported in temperate countries.

There is scarce information about the characteristics of urticaria in tropical countries, where infections and especially parasitic infections are widespread and their immunopathology is of great importance [142]. There is evidence that parasitic infections may sensitize the host and the association of AU or CU with infection by *Giardia lamblia*, *Fasciola hepatica*, *Toxocara canis*, *Echinococcus granulosus*, *Strongyloides stercoralis*, *Hymenolepis nana*, *Blastocystis hominis*, *Ascaris lumbricoides* and *Anisakis simplex* has been reported, suggesting that parasitic infections should be considered as an important cause of chronic urticaria [143, 144]. Nenoff et al. observed that treatment with metronidazole or tinidazole successfully improved pruritus in three patients who had chronic urticaria or pruritus and asymptomatic intestinal infection with *Giardia lamblia* [145]. Other investigators, believing that this parasite is an important urticaria-inducer, conducted a clinical trial of immunotherapy with *Giardia* extract in patients with CU sensitized to *Giardia* [146]. Additionally, it was observed that patients with urticaria were significantly more likely to have ≥ 5 *Blastocystis hominis* organisms per field on microscopic examination of feces [147]. In a study carried out in Egypt, Fakkar et al. investigated the association of *B. hominis* with urticaria. In total, 54 patients with urticaria and 50 controls were enrolled; the parasite was detected in a significantly higher number of patients as compared to the control group. The amoeboid form was found in 60.6 % of *Blastocystis*-positive patients with urticaria, but in none of the healthy controls [148].

In summary, urticaria is a common problem in the Tropics and seems to be clinically similar to that in temperate zones. Risk and triggering factors are probably different in the Tropics but as the information is so scarce, it is not possible to conclude. Prospective studies and well-structured research are obviously needed, particularly to define the role of parasites as risk factors [149].

### Papular urticaria

Allergic reaction to stinging insects can be immediate (IgE-mediated) or delayed (24–48 h) with predominance of cellular components. Among clinical conditions related to insect bite hypersensitivity, Papular Urticaria (PU) is a common manifestation in the Tropics. PU is an allergic and chronic skin disease caused by insect bites, with fleas and mosquitoes being the most common causal agents. PU usually appears during the first year of life; normally it improves by the age of seven years, but there are exceptions that persist until adulthood [150]. This is one of the most particular allergic tropical problems although it can be observed in any place where causal insects are present.

#### Clinical manifestations

**Fig. 1:**
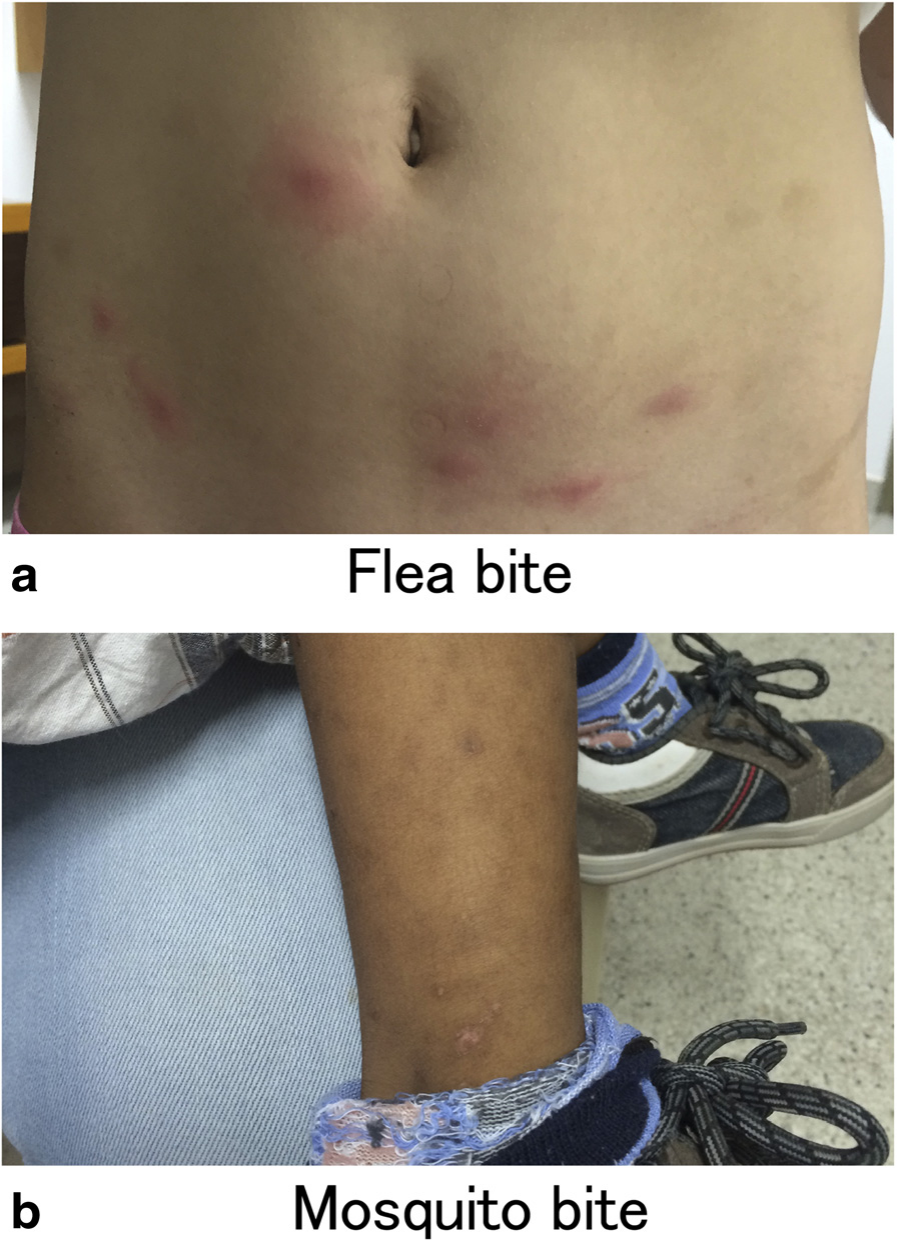
Skin lesions in papular urticaria. **a** Crops of erythematous papules induced by flea bites in a patient living in Bogotá, Colombia. **b** Hyperchromatic residual pigmentations in legs after resolution of mosquito bite-induced papules in a patient from Cartagena, Colombia. Covered body areas (i.e. trunk and back) are more prone to flea bites. Mosquito bites predominate in exposed areas, such as legs, arms and neck

The disease is characterized by a delayed hypersensitivity reaction such as papule-type skin lesions, which is the most common, or wheals, vesicles, blisters and scabs. Patients might develop hypo or hyper chromic residual pigmentations in the skin; moreover, it produces intense pruritus, severe secondary infections and scarring [150]. Hudson et al. carried out a study on a group of subjects with clinical background of papular urticaria, who were bitten on the forearms by fleas of different species *(C. felis, P. irritans* and *P. simulans*) and observed for 15 days. Twenty minutes after bite a wheal appeared (with or without erythema) and was classified as an immediate hypersensitivity reaction. After 24 h, the reaction changed to papules or vesicles and was considered a delayed response. In some cases, a bullous eruption developed after 24–48 h [151]. Similar results were obtained from Japan with mosquito bites: 65 % of patients presented delayed response (with or without immediate response); 29 % presented immediate reaction only and the remaining 6 % did not react [152]. Mosquitoes produce lesions in different areas such as the face, limbs and exposed areas of the trunk. On the other hand, lesions produced by fleas are more commonly located in pressure areas such as the waist, under the socks and in the extremities (Fig. 1).

#### Causal insects

**Fig. 2:**
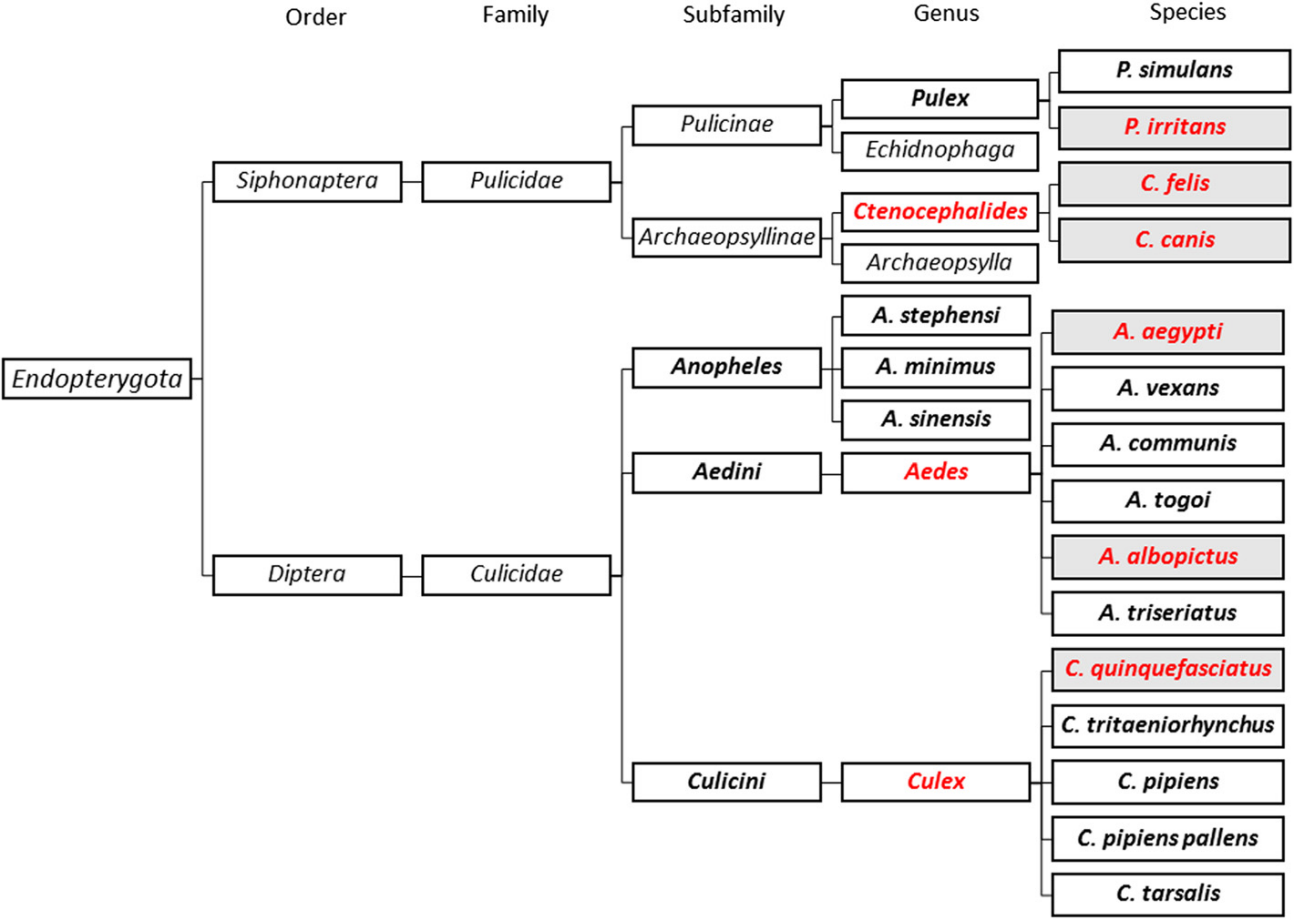
Phylogenetic tree of fleas and mosquitoes. In red the most frequent species that bite humans. Fleas group includes 2.574 species. More than 30 genera and 20.000 species of mosquitoes are distributed worldwide

More than 3000 species of mosquitoes are distributed worldwide [153]. Some of these are clinically relevant including *Aedes aegypti, Aedes vexans* and *Culex quinquefasciatus* [154–156]. Adult female mosquitoes require blood-feeding to produce eggs. In this process, they bite and inject their saliva before sucking their victim’s blood [157]. Mosquito saliva contains various substances like lysozymes, antibacterial glucosidases, anticoagulants, antiplatelet aggregating factors and vasodilators [158–162]. Fleas are obligate blood sucking ectoparasites. This group is composed of 2.574 species belonging to sixteen families (Fig. 2), but only a small number are closely associated with humans [163]. Most fleas of medical importance, such as *Pulex irritans* (known as the human flea) and the cat flea, *Ctenocephalides felis,* belong to the Pulicidae family [164, 165]. Other species related to humans are *Ctenocephalides cani*s, usually known as the dog flea but can attack other animals and humans; and *Tunga penetrans,* usually found in the sand. Species more often involved in human infections are *Ctenocephalides felis*, *Ctenocephalides canis* and *Pulex irritans* [166]. Fleas have great capacity to adapt to different home environments. The lifecycle is temperature dependent and ranges from 14 days at 32 °C to 130 days 13 °C [167]. In temperate countries, mosquito season starts slowly in the spring, when warm weather brings out the first of the bugs, peaks in summer and tapers off into fall [168]. In the Tropics, optimal conditions for mosquito survival are perennial. In India variations in the frequency of PU according to the season were found, being most frequent (16.7 %) in rainy season [169]. Burton et al. [170] reported that fleas are more common in temperate countries and mosquitoes in tropical countries.

#### Prevalence

There are several studies about the frequency and prevalence of PU in the Tropics. In Mexico City, a frequency of 16.3 % was found [171]; which decreased to 2.24 % twenty years later [172], probably because of improvement in sanitation, housing, and socioeconomic conditions. In Venezuela, Mendez et al. [173]; after dermatological evaluation of 177 Venezuelan children from a rural area, showed that PU was the commonest skin disease (25.4 %). Sanchez- Borges et al. [174] reported that PU was present in 39 % of children and 9.9 % of adults with acute urticaria attending an allergy outpatient clinic in Caracas, Venezuela. In Brazil 9 % of 19.410 cases attending an outpatient clinic in Curitiba were diagnosed as PU [175]. In Colombia, Chaparro et al. developed the first population-based study to determine the prevalence of the disease in Bogota Colombia, they noted that the prevalence of PU caused by flea bite was 20.3 % (95 % CI: 18.2 to 22.5 %) [176].

In Africa PU is regularly seen among schoolchildren, especially in countries with hot and humid climate. Studies carried out at the Dermatology Center of Bamako in Mali, reported that 6.8 % of 3479 children had PU [177]. In Nigeria, a prevalence study of skin disorders was conducted in a public primary school, including 529 (49.6 %) boys and 537 (50.4 %) girls with a mean age of 8.8 ± 2.5 years, PU was diagnosed in 35 children (3.3 %) and fungal infections and scabies were the most common skin diseases, whereas allergic conditions were nearly absent [125]. In a cross-sectional, population-based survey done in Ghana, Gabon, and Rwanda [178], the prevalence of skin diseases was estimated on the basis of physical examination by at least one dermatologist; 4.839 schoolchildren were seen and the PU prevalence was 1–2 %. In Brazzaville, Congo, a region located near a watercourse, PU prevalence studies were conducted at 100 and 500 m from the river [179], reporting a higher frequency near water and in children 6 to 8 years old. In India, a 5.2 % prevalence of PU in 2100 children was found, attributed to the tropical weather conditions in this coastal area [180]. In Pakistan, a case-series reported in 2007 where individuals of all age groups and gender having definite history of insect bites were included in the study. Out of 14.019 subjects who visited the Dermatology Outpatient Department of the Combined Military Hospital, Abbottabad, during the study period, 280 (2 %) patients were diagnosed as having PU [181].

#### Immunopathogenesis

Most of the information about immunological mechanisms associated to PU comes from the flea bite disease; histopathology suggests the involvement of an allergic component since an inflammatory infiltrate of eosinophils and lymphocytes is common, a pattern also found in biopsies from Colombian patients [182]. Interestingly, 80 % of patients had negative skin prick test to flea extract, probably because a somatic extract was employed and most allergens involved in this type of hypersensitivity are found in the insect’s saliva [183]. It should also be mentioned that in patients with reactions to mosquito bites as well only 17.6 % had positive skin test to this source [184]. A Th2 polarization of cellular immune response was observed in patients with PU induced by flea bite, given that after a polyclonal stimulation of peripheral mononuclear cells, a greater percentage of CD4^+^ IL-4^+^ T lymphocytes was detected when compared to healthy subjects, while the proportion of positive IFN –gamma T lymphocytes was significantly reduced [185].

Some flea extract components, such as 31–35 kDa, are more frequently recognized by serum specific IgE from PU children. Furthermore, components between 16 and 20 kDa were only recognized by patient sera [186]. In addition, higher basophil activation was found in children with PU when tested with fractions of different molecular weights from the flea body extract [187]. Different studies have been done to assess the role of IgE in mosquito-induced PU. Reunala and coworkers did not find significant differences in the IgE-binding frequency between PU patients and control subjects coming from tropical areas (Kenya and Mexico); which contrasts with the results observed in Finish patients displaying hypersensitivity reactions to mosquito bites, where a statistical difference in the response between patients and controls was very clear [188–190]. Another study performed in Egypt showed significant differences in the IgE recognition of bands from extracts of *Pulex irritans* and *Culex pipiens* [191].

Regarding IgG responses, frequency of band recognition by the IgG_3_ isotype was significantly greater in the control group. In addition, the humoral response profile changes in a time dependent manner. Patients having more than 5 years from the beginning of the symptoms show IgG_3_ reactivity rates which are similar to the control group, especially for proteins between 31 and 35 kDa [186]. There is an increasing interest in evaluating purified, mainly recombinant antigens and allergens involved in PU. There are several mosquito recombinant allergens causing immediate IgE hypersensitivity reactions in humans [192–194], as well as molecules of cat flea associated with allergic dermatitis in dogs [195], but there are no reports about their involvement in PU.

In summary, PU is an important allergic problem in the Tropics and causes great morbidity, especially in children. The immunopathogenesis of this condition involves mainly a cellular inflammatory infiltrate that seems to be guided by a Th2 response. The role of IgE in the pathogenesis is not clear. Allergens from mosquitoes and fleas are not completely characterized.

### Anaphylaxis

Recent guidelines on anaphylaxis have proposed that the elements that facilitate the initiation of these severe reactions are similar worldwide [196]. In this section we will present a brief description of the most common risk and aggravating factors for anaphylaxis and will discuss some characteristic aspects observed in tropical and developing countries. We will consider age, physiologic state; presence of concomitant diseases; concurrent use of medications and other cofactors that amplify anaphylactic reactions, as well as certain clinical expressions peculiar to the Tropics (Table 3).

**Table 3:** Risk factors for anaphylaxis in tropical settings

Factor	
Age	• Children: foods, drugs, insects, asthma • Adolescents: risk behavior (alcohol, drugs) • Adulthood: drugs, foods, insect venoms
Physiological status	• Perimenstrual • Pregnancy: drugs, latex
Concomitant diseases	• Asthma • Atopy • Cardiovascular diseases • Mastocytosis
Use of concurrent medications	• Beta blockers • ACE inhibitors • Calcium channel blockers • Angiotensin receptor blockers • Diuretics • Nonsteroidal anti-inflammatory drugs
Miscellaneous	• Exercise • Emotional stress • Acute infections • Fever • Alcohol • Disruption of routine • Pollinosis • Increased tryptase concentrations • Poor hygienic conditions • Weak health systems • Low educational level of the population • Poor adherence to management guidelines

#### Age

There are specific characteristics of anaphylaxis that differ according to patient’s age. For example, in infants some foods such as cow’s milk, eggs, peanuts, nuts, fish/seafood, fruits, and soy are frequently involved [197]. In the Latin American Survey on anaphylaxis 31 % of patients were younger than 6 years; and foods (36.1 %), drugs (27.7 %), and insect stings (26.2 %) were the most common offenders [198]. The presence of asthma determines a higher severity of the episodes [199]. In adolescents, the risk taking behavior increases exposure to etiologic agents related to anaphylaxis [196], while in aged people drugs, foods, and insect venoms are often involved [200–202]. Women are particularly prone to suffer anaphylaxis during the perimenstrual period. Also pregnant women are especially susceptible during delivery due to allergic reactions to drugs (penicillin) or latex.

#### Concomitant diseases

Severe or uncontrolled asthma and other chronic respiratory diseases [203], cardiovascular diseases [204], and mastocytosis [205, 206] are commonly observed in patients who develop anaphylaxis. In Latin America asthma is present in 41.9 % of children with anaphylaxis [198]. However, regarding drug-induced anaphylaxis (DIA) a recent study carried out in Latin America did not identify any specific host risk factors [207]. In fact, asthmatic and atopic patients presented less severe DIA reactions, an observation that contradicts other studies where asthma and AD were associated with a significantly greater risk of anaphylaxis [208].

In Brazil, Aun et al. observed a high frequency of atopy and asthma in patients with DIA, although those conditions were not associated with the severity of the reaction. Also, a previous hypersensitivity drug reaction with the same drug was present in 15 % of patients with DIA [209]. In children with anaphylaxis repeated attacks occurred in 42.0 % [198]. These observations emphasize the need for adequate patient’s and physician’s education in order to prevent undesirable recurrences.

#### Concurrent medications

The intake of beta blockers, angiotensin converting enzyme inhibitors, calcium channel blockers, angiotensin receptor blockers and diuretics can increase the risk of anaphylaxis, and this association is especially frequent in old people [202]. In addition, nonsteroidal anti-inflammatory drugs (NSAIDs) have been shown to enhance anaphylaxis [210]. This observation has to be taken into account especially in developing countries where NSAIDs are easily obtained without a medical prescription.

#### Other cofactors that amplify anaphylaxis

A number of other elements have been incriminated as exacerbating factors of anaphylaxis [210]. They include exercise, emotional stress, acute infections, fever, concomitant ingestion of ethanol, disruption of routine, and pollinosis [197]. For hymenoptera venom anaphylaxis, some contributing factors for increased severity have been identified, including elevated baseline tryptase concentrations, older age, absence of urticaria and angioedema during anaphylaxis, and symptom onset within 5 min after a sting [211]. Due to the climatic conditions, the Tropics favors insect living and therefore reactions to stings are more likely to occur in these areas of the world.

#### Anaphylaxis from hidden food allergens

Associated with the poor hygienic conditions prevalent in tropical countries, allergic reactions to allergens hidden in the foods are more common in the Tropics. An example is the atypical anaphylaxis induced by the ingestion of mite-contaminated foods (oral mite anaphylaxis [OMA], pancake syndrome) that has received increased attention in recent years [212]. Climatic conditions in the Tropics, with high temperatures and humidity, are favorable for mite growth, determining easier contamination of foods, especially wheat or corn flours. Eventually some atopic mite-sensitive patients will develop systemic symptoms when exposed to mite allergens through the oral route [213]. Improved sanitation and some prophylactic measures have been proposed to decrease the risk of OMA [212].

#### Anaphylaxis and helminths

Anaphylaxis may occur in persons infected with *Echinococcus granulosus,* and especially when fluid from the ruptured cysts is released into the circulation in patients who produce specific IgE to the parasite. Also, rare cases of anaphylaxis triggered by *Taenia solium* and *Ascaris lumbricoides* have been published [214]. *Anisakis simplex* present in raw or undercooked fish can also cause anaphylaxis, and this is more frequent in countries where the use of raw fish is common [215].

Anaphylaxis is under-recognized and undertreated everywhere in the world. Also, emergency treatment of reactions is deficient in many countries, with low rates of utilization of epinephrine, and higher use of second-level drugs such as corticosteroids and antihistamines. For example, in the Latin American Online Survey on anaphylaxis the rate of utilization of adrenaline was as low as 34.6 to 37.3 % [198, 216], whereas for DIA it was less than 30 % [207]. Educational campaigns for physicians and patients should be encouraged in order to improve the management and prevention of anaphylaxis since pitfalls are common [217]. Referral to specialists in allergology should be promoted for diagnostic orientation, and implementation of preventive measures that include immunotherapy and immunomodulation.

In summary, risk factors for anaphylaxis in the Tropics are patient’s age, high prevalence of asthma, abuse of alcohol, recreational drugs and medications, drug self-prescription, high rates of infections, stress from economic and political instability, and the high concentration of environmental allergens (mites, pollens from abundant flora, molds, insects), pollution, and the presence of hidden allergens in the food.

### The impact of allergen sensitization on allergy symptoms

The relationship between allergen sensitization and allergic symptoms has numerous aspects that are currently under investigation. It is indeed one of the central interrogates of Allergy. Although intuitively logical that the stronger the sensitization the more probabilities of inducing symptoms, the problem is more complex and includes the conjunction of several predisposing and protecting factors related to the environment and the genetic background. However, since sensitization patterns, as well as other situations, are very unique in the Tropics, their study could help understanding the natural history of allergy and direct future research. In the following three sections we present the type and effects of sensitization on allergy symptoms under the particular conditions of the Tropics.

### Allergen sensitization and asthma symptoms

Sensitization to aeroallergens is an important feature of atopic asthma and impacts on long-term management which includes environmental control and allergen immunotherapy. Knowledge of the fauna and flora provides information on relevant environmental allergen exposure in the population. This is limited in the Tropics and other parts of the world, where a complete catalogue of clinically relevant allergens is missing. A World Atlas of Aeroallergens has been developed by the World Allergy Organization (WAO) to guide clinical efforts and research in the field of aeroallergen sensitization. Here some particular characteristics of allergen sensitization in tropical/subtropical regions are discussed.

#### Indoor Allergens

Numerous population and clinic-based cross sectional studies have shown that sensitization to indoor aeroallergens is significantly associated with childhood asthma in the tropical region of all continents [58, 59, 110–113, 115, 218–237]; summarized in Additional file 3: Table S3. House dust mites are by far the most predominant allergen sources in the Tropics where populations are perennially exposed, mainly to *B. tropicalis* and *D. pteronyssinus* [238]. Since it thrives in warm and humid climates, *B. tropicalis* is an important allergen in tropical and subtropical regions [13, 59, 239–241]. The HDM fauna in Los Baños, Laguna, Philippines included 32 species of mites belonging to 7 families. *B. tropicalis* (265 mites/g of dust in 87 % of households) and *D. farina*e (71 mites/g of dust in 58 % of households) were the most prevalent and abundant species [242]. Samples of house dust collected from 50 homes in El-Arish city, North Sinai, Egypt, revealed that 34.6 % of the samples had mites [243]. Blo t 5 allergen was detected in 34 out of 88 raw and processed food samples including wheat, corn, rice, bean, wheat and corn flour, cake, and rusk collected from retail stores in Benha city in the Nile Delta of Egypt [241]. In some tropical areas, the majority of the homes from mite allergic patients with asthma and/or rhinitis present concentrations of group 1 allergens >10 μg/g of dust in at least one site of the house [244–246]. Although sensitization to *B. tropicalis* is very common, levels of *B. tropicalis* allergen Blo t 5 have been consistently low in homes of allergic patients in Brazil [247, 248]. It has been reported that specific IgE antibodies to *D. pteronyssinus* correlate with total serum IgE levels in atopic asthmatics suggesting that they contribute to the IgE levels [249].

Sensitization to these two mite species is very prevalent in children with asthma in tropical Asia, Central and South America, and Africa, with rates as high as >90 % in some studies. *B. tropicalis* expresses species specific allergens that have little or no cross reactivity with *Dermatophagoides* spp. [250, 251]. In Singapore and Taiwan the major allergens of *D. pteronyssinus* associated with childhood and adult asthma are Der p 1 and Der p 2, and for *B tropicalis*, Blo t 5 [218, 252]. IgE response to Blo t 1 was 72 % for sera from patients with positive skin test to *B. tropicalis* and cross-reactivity with *D. pteronyssinus* was not detected [253]. Blo t 21, a 129-amino-acid protein, in spite of sharing 39 % identity with Blo t 5, was found to be a non-cross reactive major allergen [254]. These two components have low IgE cross-reactivity with *A. lumbricoides* antigens; which would therefore confer higher specificity in serodiagnosis assays than the crude mite extract [255].

Although not involving children but mainly university students in Singapore, Andiappan et al. reported that IgE sensitization in asthma and allergic rhinitis was almost exclusively to *D. pteronyssinus* and *B. tropicalis* [60]. More than 80 % of the cohort was sensitized and the levels of specific IgE were 8 to 30 times higher than those to other non-HDM allergen included in the study (German cockroach, cat and dog dander, Bermuda grass pollen, and a panel of molds). The HDM specific IgE levels also correlated with serum total IgE. The authors concluded that the allergic response in airway allergy is dominated by a single class of allergen source, dust mites. This is also an exclusive feature of asthma and respiratory allergies in urban populations of the Tropics. In comparison, assessment of respiratory allergy in the temperate climates would involve a panel of indoor and outdoor allergens, and correlation of allergen specific IgE titers with total IgE levels would only be at most weak [256].

Relatively high rates of sensitization to other dust mites, such as *Euroglyphus maynei, Angiostrongylus malaysiensis*, *Sturnophagoides brasiliensis*, *Tyrophagus putrescentiae* [225], *Lepidoglyphus destructor* and *Aleuroglyphus ovatus* [257] have been associated with asthma. A study in Quito (Ecuador) detected large populations of house dust and storage mite allergens in areas above 2500 m of altitude, where humidity remains high year round. Positive skin prick tests (SPT) reactions to *D. pteronyssinus, D. farinae, B. tropicalis, L. destructor, T. putrescentiae, A. ovatus, A. siro, and G. domesticus* were obtained in 60.9, 56.8, 17.0, 19.3, 10.6, 15.8, 8.8 and 11.0 % of the patients, respectively [258]. The prevalence of positive SPT to different mite species was determined in asthmatic adults and children living in seven cities of five Latin American countries. Sensitization to *D. pteronyssinus* varied from 60.7 % in Cartagena to 91.2 % in São Paulo and to *B. tropicalis* from 46.5 % in Mexico City to 93.7 % in São Paulo. In general, skin sensitivity to storage mites was lower than to domestic mites. Reactivity to mite allergens is very common in young and adult asthmatics in Latin America, in areas both at sea level and at high altitudes reinforcing preventive measures in the treatment of asthmatics in Latin America [257]. Of 176 atopic patients from Caracas – Venezuela, sensitization to *D. pteronyssinus* was present in 97.2 % of the patients and sensitivity to *B. tropicalis* in 91.6 % [59].

The prevalence of sensitization to *D. pteronyssinus, D. farinae and B. tropicalis* in 1500 Taiwanese primary school children were 90.79, 88.24 and 84.63 %, respectively [259]. In tropical Singapore, the most common mite allergens recognized in atopic children were Der p 1 (64 %), Der p 2 (71 %), Blo t 5 (45 %), Blo t 7 (44 %), and Blo t 21 (56 %) [57]. Among Malaysians, older children had significantly higher sensitization rates to *D. pteronyssinus* (63.3 % vs. 23.1 %) and *B. tropicalis* (63.2 % vs. 16.7 %) compared to younger children [49]. A study from Kolkata, India revealed SPT positivity rates to *D. pteronyssinus* (75 %), *B. tropicalis* (72 %), and *D. farinae* (63.7 %) in 1079 asthmatic patient. The high sensitization rate to *B. tropicalis* suggested a causal role in airway allergy [260]. *D. pteronyssinus* and *B. tropicalis* represented an important source of storage mite in Haikou, a tropical island in Southern China, but *D. farinae, L. destructor*, and *A. siro* allergens were found in very few environmental samples. The sensitization of asthmatic children to *D. farinae* was considered to represent cross-reactivity with *D. pteronyssinus* and *B. tropicalis* [261].

In Africa, mite fauna followed the same patterns of other tropical/subtropical regions. Sensitization to *D. pteronyssinus*, *D. farinae* and *B. tropicalis* was found in 53.2, 49.8 and 47.8 % of patients enrolled in a cross-sectional study in Yaounde, Cameroon in sub-Saharan Africa [262]. In the same city, the Odds ratios (95 % CI) for sensitization of a group of adolescents to *D. pteronyssinus, D. farinae* and *B. tropicalis* in relation with asthma were 7.28 (3.75–14.15), 2.65 (1.27–5.45) and 3.23 (1.68–6.21), respectively [263]. Sensitization to one or more mite species was reported in 24 % of Cairene children with allergic diseases; *D. pteronyssinus* and *D. farinae* were the most common sensitizers [264].

Besides dust mites, sensitization to cockroach allergens is the next common risk factor associated with asthma in the Tropics [90, 110, 112, 115, 218, 220, 229, 230, 36]. The rates of sensitization to *Periplaneta americana* is slightly higher than *Blattella germanica* in Asia and South America when allergens of both sources were assessed [110, 223, 225, 230]. However, data from Singapore and south China (Guangdong) have shown that the majority of asthmatics who were sensitized to cockroach were also sensitized to house dust mites [60]. Cross inhibition studies using sera of subjects from south China suggests that *D. pteronyssinus* was the primary sensitizer for cockroach sensitization in their population. Discrepancy between the frequency of IgE sensitization to cockroach and the level of its allergens in house dust has been reported. Cross-reactivity due to tropomyosin, present in mites, cockroach, shrimp and helminths may be one explanation for this finding in areas where they are common [227]. Cockroach allergy is important in Brazil and other places of South America, yet environmental levels of cockroach allergens have been surprisingly low in several studies [244, 265, 266]. The reasons for this finding are not completely understood. Skin test reactivity may be due to IgE responses to cross-reactive allergens in mites [266].

Skin test to at least one cockroach extract was positive in 83.1 % of atopic patients from Caracas, Venezuela [59]. In Thailand, the incidence of cockroach allergy by skin testing using crude extract ranged from 41 to 77.5 % [267, 268]. The results of IgE-immunoblotting have shown that 3 of 18 (16.7 %) allergic sera gave a positive reaction to the native troponin-T preparation of *P. americana* implying the role of this muscular regulated protein component as a minor allergen in Thais [269]. Cockroach allergens were detected in 93 % of households in Guangzhou city, southern China, and were higher in living room than in bedding samples [270].

Cockroach sensitization was detected in 43 out of 51 (84 %) asthmatic Egyptian children from Cairo city, seven of them with high specific antibody response [271]. In Lagos, Nigeria, 44.6 % of a group of asthmatic patients had positive skin tests to cockroach allergen compared to 9 % of the control subjects [272]. SPT for *B. germanica* was positive in 47 (25.5 %) patients from Yaounde, Cameroon [273]. In the South African Free State, 38 % of a group of allergic rhinitis patients were sensitized to *B. germanica* as tested by ImmunoCap [274]. Sensitization to cockroach allergens was an independent risk factor for asthma in Ghanaian children on multivariate analysis (OR, 4.9; 95 % CI, 1.3–18.6) [115].

Inhalant allergy to insects other than cockroaches is also present in the Tropics; sensitization has been reported to the mayfly, housefly, caddis fly, moth and ant [275]. The housefly (*Musca domestica*) was found to be an important allergen in children with asthma in Northern India; the sensitization rate was 37.6 % [276]. A high frequency of sensitization to a silkworm moth (*Bombyx mori*) was observed in atopic children and adolescents with respiratory allergies from the city of Curitiba, state of Paraná, Brazil. The extract prepared from the wings of this moth species was effective in demonstrating this sensitivity [277]. The major allergen of *B. mori* larvae (Bom m 1) is constituted by arginine kinase protein and displays cross-reactivity with cockroach arginine kinase. There is also evidence suggesting that mosquitoes could be inducers of respiratory allergy [278]. Finally, anemophilous indoor fungi in tropical regions have consistently determined a low degree of IgE sensitization in respiratory allergic patients [279, 280].

#### Outdoor allergens

Sensitization to outdoor allergens (Additional file 3: Table S3), such as pollens and molds, has been reported in tropical environments; however, rates do not reach the levels observed in patients with seasonal asthma and allergic rhinitis in temperate climates. This could be due to lack of exposure, but limited knowledge about the native flora should also be considered. Although numerous studies have described tropical flora, the information about the allergenic properties of pollens and molds is scarce. Sensitizations to Oil Palm pollen (40 %) and Resam fern spores (34 %) in Singapore [226] were associated with atopy. Similar but lower rates of sensitization were reported in Indonesia [219] and Thailand [220].

The subtropical grasses *Chloridoideae* (e.g. *Cynodon dactylon*; Bermuda grass) and *Panicoideae* (e.g. *Paspalum notatum*; Bahia grass) species are abundant in regions of Africa, India, Asia, Australia and the Americas [281]. In Brazil, *Lolium multiflorum*, also known as Italian rye-grass, is the main cause of pollen allergy [282]. However, other allergenic grass species grow randomly in city suburbs and on abandoned plots of land, such as *Anthoxanthum odoratum* (sweet vernal grass), Bermuda grass, *Holcus lanatus* (common velvet grass), *Paspalum notatum* (Bahia grass) and *Bromus spp.,* among others [283].

*Dolichandrone platycalyx*, commonly known as Nile trumpet tree, is believed to have originated in East Africa. The tree mostly grows in tropical climates, but temperate species are also found. *D. platycalyx* was the fourth most common sensitizer after *Parthenium hysterophorus*, *Prosopis juliflora,* and *Artemisia vulgaris* in Karnataka state, India. It was found to be a moderate pollen producer [284]. *Catharanthus roseus* or periwinkle plants are widely grown as garden plants in the Tropics and subtropics. In an agricultural farm in the suburban zone of Calcutta, India, *C. roseus* pollen was found to be almost perennial with 3.6–5.4 % contribution to the pollen load. Positive SPT reactions were detected in 29.8 % of 282 patients with respiratory allergy [285]. IgE mediated hypersensitivity varied from 4.40 to 13.20 % in Indian atopic subjects to pollens of one or more species of *Brassica*, a source of Rapeseed-mustard [286].

*Caryota mitis* is also a common plant in tropical and subtropical areas. It produces large amounts of pollen, which has great potential for allergenicity in the pollination season. A study from Singapore sought to investigate the components of *C. mitis* pollen contributing to human allergic diseases. Twelve out of 20 serum samples positively reacted to recombinant CmProfilin, as shown by Western blotting and ELISA [287]. Sensitization rates to ragweed and Japanese hop in South Korean children increased annually (ragweed, 2.2 % in 2000 and 2.8 % in 2002; Japanese hop, 1.4 % in 2000 and 1.9 % in 2002) [288]. Children living in Changhua area of Taiwan had a higher pollen sensitization rates (Bermuda grass, 17.2 %; Timothy, 12.3 %; ragweed, 5.7 %) than cat or dog dander sensitization rate [289]. Weeds are the most common aeroallergens in Ahfaz, Khuzestan province, which is a tropical region in Southwestern Iran. It represented the most prevalent outdoor aeroallergen (89 %) followed by tree and grasses [290]. Maize pollen extract was identified as a potential aeroallergen in Iran belonging to group 13 of allergen categories [291]. In some parts of Saudi Arabia, species of *P. juliflora* have been introduced by the millions as roadside ornamentation. SPT positivity ranged between 11 and 76.1 % in 4 sites in the country. Multiple sensitivities to other pollen antigens were detected in all patients. The level of airborne *Prosopis* pollen detected in Gizan exceeded 90 grains m^3^ of air [292].

#### Fungi

In some tropical environments, such as Puerto Rico and Saudi Arabia [293, 294], basidiospores are the predominant fungal particulates in the atmosphere However, their contribution to allergic respiratory diseases has not been extensively studied as has occurred with mitosporic fungi. Sensitization rates to crude spore extracts of the basidiomycetes *Ganoderma applanatum, Chlorophyllum molybdites* and *Pleurotus ostreatus* were reported in 30, 12 and 12 % of 33 allergy patients from Puerto Rico [295]. In asthmatic children from the same country exposure to the highest quartile of glucan, a component of the fungal cell wall that is used as a marker of fungal exposure, was associated with more visits to the emergency room (95 % CI for adjusted odds ratio, 2.7–28.4; *P* < 0.001) [296]. In Cartagena, Colombia, sensitization to *Penicillium notatum* was 28.3 % in asthmatic patients in a study where sensitization to house dust mites ranged between 54 and 74 % [297].

#### Helminths

In tropical regions the coexistence of allergic asthma and helminthic infections (two Th2 biased immunologic conditions) is well documented; therefore IgE sensitization to species-specific and cross-reacting helminth antigens and its impact on allergy symptoms should be analyzed and will be discussed in other sections of this review.

### Sensitization to common food allergens

Food allergy (FA) is a good example of how nature, cultural patterns and lifestyles influence the IgE responses and allergic symptoms. Its occurs frequently in children under three years of age (3–15 %), but also in older people (6-8 %) [298]; although it seems that in the Tropics the same pattern occurs, more cohort studies are needed to evaluate the natural course of this problem. Studies in North America and Europe suggest that in children more than 90 % of reactions are induced by a small group of foods, mainly egg and cow’s milk, while in adults the fish and shrimp are more frequent. Soy, wheat, tree nuts and peanuts are also a common in these populations [299]. Recent studies in tropical and subtropical regions in Asia, Africa and Latin America suggest that other foods have equal or higher frequency of sensitizations than previously described [65, 300, 301]. In addition, analyses of the role of anaphylaxis triggers show that food is an important cause of severe allergic reactions in Asia [302] and in Latin America [303], being fruits primarily involved in tropical countries such as Singapore, Mexico, and South of China.

Several surveys suggest that peanut allergy is less common in populations living in tropical regions of Asia [302, 304]. Furthermore, the patterns of FA appear to be different than those observed in temperate climates. For example, shellfish allergy predominates in both children and adults, with prevalence rates higher than those reported in temperate countries [304, 305]. Besides genetic and ethnic factors [306], these features of food allergy are likely related to environmental immunomodulatory factors as well as exposure to aeroallergens. The age of introduction of foods is another factor that seems to be important at least to peanut allergy [307] and could explain why this allergy is less common in some tropical populations. However, cohort studies specially designed to explore this aspect in the Tropics are still missing. Food allergen sensitization has been evaluated in several surveys but a direct comparison of data is difficult as the clinical characteristics of subjects are heterogeneous. However, in this section we analyze cross sectional or case control studies focused on children with various allergic conditions, such as AD, asthma and FA. Most of the studies are from tropical and subtropical Asia [114, 259, 279, 289, 308–317] and are summarized in Additional file 4: Table S4.

In general, egg and/or cow milk are the predominant allergens [318]; but there are exceptions. Sensitization to shellfish such as shrimp/prawn and crab is also common, especially in older children. For example, in Hong Kong [309], Taipei [259], Malaysia [310] and Singapore [311], sensitization to these allergen sources was either ranked top or second to egg when school aged children were surveyed. This high rate of sensitization mirrors the relatively high rates of reported shellfish allergy in the region [304] and the frequency of cross reacting allergens from other sources. Tropomyosin has been the most often implicated as the cross reactive allergen. This is supported indirectly by IgE inhibition studies using sera of Spanish shrimp allergic subjects living in a warm and humid climate [319].

Sensitization to soy and wheat are the next frequently reported allergens. Although not systematically studied in the Tropics, IgE mediated soy allergy is perceived to be less common compared to the frequency of sensitization [320]. Similarly, wheat allergy appears to be uncommon, with the exception of Thailand where severe wheat allergy has been described [321]. The high rates of sensitization to soy and wheat have not correlated with true allergy. Unlike allergies to cow milk, egg and peanut, where high levels of specific IgE are useful predictors of a positive oral food challenge, soy and wheat specific IgE levels are poor predictors of true allergy [322]. The sensitization to soy and wheat observed in the Tropics and subtropics is also likely to reflect this phenomenon. The high rates of asymptomatic sensitization may be due to cross reactive allergens, possibly cross reactive carbohydrate determinants, which have poor correlation with clinical allergy.

Although the prevalence of peanut allergy is relatively low in the tropical region compared to temperate climates, sensitization to peanut shows importance in some studies from Hong Kong, Singapore and Brazil. This relative high prevalence of peanut sensitization may be asymptomatic and also associated with cross reactivity to environmental allergens. A study involving Ghanaian schoolchildren showed that IgE sensitization to peanut (17 % in the cohort) was associated with *S. hematobium* infection and was not associated with clinical peanut allergy [317]. The authors postulated that carbohydrate cross reactive determinants accounted for this cross reactivity as skin prick test positivity and reported adverse reactions to peanut were far lower than sensitization detected by IgE testing, at 2 % and 1.5 % respectively. In contrast, sensitization to Ara h 1 and Ara h 2 major allergens was common in cases of true peanut allergy in Singapore [323] and Thailand [324]. This profile is similar to that found in peanut allergic subjects in the US [325]. In Ghana, a study of food allergy in 1407 school children found that 11 % had adverse reactions to foods but only fifty percent of these children showed a positive SPT reaction mostly directed against peanut and pineapple [326].

In a review of FA literature in Latin America, 41 reports were original contributions [66]. In Mexico City [327] food sensitization as detected by skin tests was 31 %, mainly in children up to 7 years of age. Foods commonly involved were fish (12 %), milk (7.7 %) and seafood (6.5 %). In general, frequency of sensitization to egg and peanuts was lower than those reported in the US and Europe [326, 328–332]. Naspitz et al. [333] used a unified panel of foods for a case-control study in several cities of Brazil; although no food was associated with specific allergy, the presence of specific IgE was higher among patients (79 %) than controls (25.8 %) and the most frequent allergens were fish, eggs, cow milk, wheat and peanuts. In Costa Rica, Soto Quirós et al. [334] evaluated specific IgE (MAST system and Phadia) to 15 foods in 183 children with asthma and 275 controls. No significant differences on sensitization were found between the two populations. Sensitizing foods in asthmatics were fish (60 %), mixed vegetables (58 %), almonds (54 %), garlic (53 %), yeast (51 %), wheat (50 %), soybean (48 %), egg (48 %), milk (43 %), peanuts (42 %), corn (40 %), onion (38 %), orange (28 %) and cereal’s mixture (15 %). This pattern was almost the same in controls.

In general, studies about FA are difficult to compare due to differences in definitions and the food panels tested [335, 336]. Recently, J Boye [65] did a review on the epidemiology of FA in developing countries and noted that the current data were inadequate to perform a systematic review or meta-analysis. The same difficulty was found during a systematic review conducted in Latin America [66]. The heterogeneity of the trials and the lack of uniformity in the foods tested within the same study lead to significant errors when assigning the weight of each report [337].

In summary, food sensitization is a relatively common phenomenon among atopic subjects in the Tropics. This review suggests that food allergy in the Tropics has points in common with studies in other regions but also distinctive features; especially in terms of the type of food that causes sensitization. Studies with provocation tests are required to confirm the clinical relevance of these sensitizations in Latin America and Africa. Another urgent need is to perform multicenter studies using the same methodology and the same food panels, including a large number of fruits, vegetables and other foods of each tropical region [338].

### Sensitization to pets

Ownership of pets is supposedly frequent in the Tropics with subsequent exposure to their allergens. The most common pets are cats and dogs; the main allergens are Fel d 1 and Fel d 4 for cats [339] and Can f 1 and Can f 2 for dogs [340]. Controversy exists as to the implications and significance of pet ownership with regards to pet sensitization, and the development of atopic disorders [341]. The prevalence of pet sensitization in tropical countries and the relationship between pet ownership and atopic disease has not been described in detail. Here we present and analyze data pertaining to pattern, prevalence and significance of pet sensitization in children from tropical regions. Eighteen studies [105–115, 221, 225, 233, 313, 334, 333] describing pet sensitization were found. They are summarized in Additional file 5: Table S5. Thirteen were cross-sectional, almost all drawn from children seen at outpatient clinics. Only three [111, 112, 233] comprised children from community schools. All studies focused on cat and/or dog sensitization, based on skin prick tests, specific IgE assessed by ImmunoCap, RAST IgE [334] or MAST pipetting [334].

The prevalence of sensitization to pets varied widely. One report [111] describes the prevalence of cat sensitization in children; 3.7 % in Hong Kong and 4.3 % in Guangzhou, China. The authors included Beijing as a third study site in a temperate region, noting that cat sensitization rates were highest in this city. They also found that cat sensitization was associated with current wheeze in Guangzhou but not Hong Kong or Beijing; and associated with bronchial hyperreactivity in Beijing, but not in the other two cities. The remaining surveys reported prevalence of pet sensitization in children with asthma or other allergic disorders. Dog sensitization was between 0 % of children with asthma in Thailand [221] and Ghana [115] to as high as 33.9 % in children with asthma in Singapore, aged 6–14 years [225]. Rates of cat sensitization were also variable, ranging from 1.5 % in Singapore [114] to 40 % of asthmatics in Hong Kong. Sensitization to cat and dog dander occurred late in childhood, at an average age of 8.9 years in the single large cross-sectional study of children with allergic rhinitis in Singapore [105]. Cat sensitization was associated with the diagnosis of asthma in Nigeria [233] and Hong Kong [113], as well as more severe asthma in Mexico [106]. Conversely, two studies did not find any association of cat or dog sensitization with asthma [115, 334]. Dog sensitization in Costa Rica [112] was linked to eczema. None of the studies examined the relationship between pet ownership and pet sensitization, and most of them found that dust mite sensitization was more significant than any pet sensitization.

Since many factors may influence the pattern, prevalence and significance of sensitization, a comparison between the data from the Tropics and non-tropical countries is dubious, at least until larger, more robust studies in the Tropics are conducted. For example, studies based on patients attending specialist clinics introduce a selection bias into the subjects recruited. Of the three cross-sectional population studies (less likely of having selection bias) only one examined the prevalence of pet sensitization [111]. There were no cohort studies; therefore, assessing the temporal relationship between pet ownership, pet sensitization and disease was not possible. Leung et al. [108] demonstrated a correlation between cat or dog ownership and Fel d 1 levels in mattresses, bedroom and living room. This was also shown in other recent study in the Tropics which examined the association between indoor allergen levels and pet ownership, and found an association between cat ownership and level of Fel d1 in child care centers. However, the authors did not examine other relevant variables, such as pet sensitization or presence of allergic disease [342]. To our knowledge, there are no other recent studies in the Tropics that have looked at allergen levels, pet ownership and impact on the individual’s health or pattern of sensitization.

A study in rural Chilean children showed inconsistent association between animal exposures and allergy. Contact with dogs in the first year was a protective factor of rhinitis (OR 0.47, 95 % CI 0.28–0.80), but contact with poultry and cats early in life increased the risk of rhinitis (OR 1.42, 95 % CI 1.06–1.88; OR 1.82, 95 % CI 1.06–3.14) [343]. Exposure to high levels of mouse allergen was more frequent among non-recurrent wheezers [248]. A positive skin prick test was observed for cat epithelium (28.7 %) and dog epithelium (21.3 %) in 94 children from Palmas, Tocantins, Brazil [254]. In Southwest Nigeria, positive skin tests to cat, cockroach, mango blossom and mouse epithelium were more frequent in asthmatics than in healthy controls, especially in the rural communities [233].

Caution should be taken in reporting sensitization to cat in tropical areas endemic for parasites, as they can induce anti-α-gal antibodies that cause false positive binding to the α-gal moiety of the cat allergen, Fel d 5. This means that tests suggestive of positive IgE to cat may not truly reflect cat sensitization; Arkestal et al. [344] demonstrated this in parasite-infected patients in Africa who did not have cat allergy. Given that Fel d 1 is the major cat allergen in most of these patients, component-resolved testing to Fel d 1 may be necessary to avoid this diagnostic confusion in patients from parasite-endemic regions.

In summary, given the inconclusive data on pet sensitization in the Tropics, further studies are needed. Large, well-designed birth cohorts for assessing sensitization to various pets, and relate these to the specific socio demographic factors, patient comorbidities, pet ownership and level of exposure, are needed to improve our understanding of the role of pets in allergic disorders.

### Birth cohorts exploring early sensitization in the Tropics

Birth cohort studies are valuable strategies to analyze early sensitization in humans. Concerning asthma, more than 130 studies have been started around the world in the last 30 years [345]; however, few have been done in the Tropics [248, 346]. Birth cohort results about early sensitization, risk factors and its relationship with atopic disorders in the Tropics are described below.

#### Latin America

Most information about early sensitization in the Tropics derives from the FRAAT birth cohort, a follow-up of ~300 children living in underprivileged urban neighborhoods in the Caribbean coast of Colombia [46]. The genetic background of this population resulted from admixture between Native Americans, Africans and Spaniards [347]. Cord blood (CB) IgE was the earliest time point analyzed in this study. High CB total IgE was positively associated with later sensitization, a common finding in studies from temperate countries [348]. In contrast to other latitudes where high total CB IgE has been found associated with atopy-related phenotypes [349, 350], this condition was protective from recurrent wheezing in this cohort, replicating an observation from Sunyer et al. in Tanzanian children [351]. Compared to other latitudes allergen sensitization rates in this cohort were high in the first years of life [248, 352, 353], being *B. tropicalis* the main sensitizer. Interestingly, total and specific IgE increased rapidly during early life and sensitization at 3 years old almost reached the proportion observed in adults. Most important risk factors for positivity to *B. tropicalis* were poverty/unhygienic indicators which could be related to helminth infections. This cohort has a low burden of ascariasis, a condition that may potentiate Th2 responses through various mechanisms including cross-reactivity with HDM allergens [354]. Although detection rate of soil-transmitted helminthiasis by stool examination was low, it was observed by IgE serology that half of the cohort was immunized to Asc s 1 (a nematode specific marker), which is indicative of frequent parasite exposure at this period of life in this population [355].

ECUAVIDA is the largest birth cohort study in Latin America. Ethnicity of the study population has been reported as Mestizos (90 %), Afro- Ecuadorians (7 %) and Amerindians (3 %) [356]. Preliminary data presented in the cohort indicated that SPT responsiveness to at least one allergen was 17.1 % at three years old; being *Dermatophagoides spp*. the most frequent sensitizer. Sensitization to food allergens, fungi or grass was scarce. This study involves children from a rural community in Esmeraldas (Ecuador) wherein, at least 29 % of infants have been infected with STH at 2 years old [48]. Unfortunately, in ECUAVIDA the IgE response to *B. tropicalis* has not been evaluated; therefore, positive cases of sensitization could be missed. The relationship between allergen levels and early IgE responses has not been fully evaluated in Latin American birth cohorts; however, Rullo et al. showed that most children in their cohort were exposed to a high load of mite allergens (2 μg/G of Der p 1 in mattress). A positive association with sensitization was not reported; instead, they found no statistical association between allergen levels and recurrent wheezing [248].

#### Asia

GUSTO birth cohort is currently evaluating the natural history of allergic diseases in Singaporean population [357]. Cumulative prevalence of rhinitis was 32 % in the first 18 months of life; a high rate in comparison with cohorts from other latitudes [358]. Low income, eczema and familiar history of atopic symptoms were detected as risk factors for recurrent/prolonged rhinitis. As detected by SPT, only 20 % of cases were sensitized to an aeroallergen, mostly HDM; however it was more frequent among cases than controls. The PATCH study, in Taiwanese population, revealed important information about food allergen sensitization. At 1.5 years, positive specific IgE levels to common food sensitizers were detected in almost half of children, but declined with age. Early sensitization to food components was associated with AD, in contrast, to inhalant allergen sensitization that was found as a risk factor for respiratory allergy [359]. In this cohort, vitamin D levels in pregnant mothers and children (starting in cord blood serum) were measured; high correlation was found among mother’s and cord blood levels. Low maternal 25(OH) D levels were associated with allergic sensitization, AD, asthma in early childhood [360]. Another birth cohort from Taiwan also explored the relationship between AD and sensitization, finding egg white sensitization as a risk factor [361]. Accordingly, it seems that in industrialized countries from tropical/sub-tropical Asia the relationship between sensitization and allergic disorders have similarities with other latitudes. Another Asian cohort investigated a very different scenario to the previous mentioned cohorts. It enrolled pregnant mothers and their subsequently born children living in endemic villages for filarial (*W. bancrofti*) and STH in Indonesia. At 4 years old of follow-up, high rates of HDM and cockroach sensitization were found by IgE serology, but not by SPT. Sensitization to food allergens was rare. Interestingly, there were found negative associations of filarial infection as well as poverty indicators with child SPT, but not with positive serum IgE [346]. Relationship of sensitization with atopic disorders was not determined.

#### Africa

The Butajira Ethiopian cohort explored sensitization at three years old by SPT with cockroach and *D. pteronyssinus* extract. Overall sensitization rate was low (8.7 %) and unrelated to the presence of allergic manifestation at three years old. Der p 1 or Bla g 1 levels, as well as parental history of allergy were not associated with allergic symptoms or sensitization in children [234]. At a later point, incident cases of sensitization at 5 years old were significantly associated with AD or rhinitis, but no wheeze [362]. Data from this cohort revealed other important factors that may protect or increase the risk of sensitization and allergic disorders. *Helicobacter pylori* infection (at age 5) was inversely associated with all allergic outcomes at age 5 except wheeze and reached statistical significance for sensitization – an effect observed only as a trend at 3 years [234, 362]. Use of paracetamol was associated with wheeze, AD and sensitization [363], a previous finding in cross-sectional studies from temperate countries [364].

As observed in adults, HDM are the most important sensitizers in early life; meanwhile, responsiveness to food allergens is rare [346]. Sensitization to common airborne allergenic sources, determined by specific IgE, is actually higher than in temperate countries [13, 346]. However, when assessed by SPT, lower rates are found [355]. Poverty conditions increase the risk of helminthiases as well as early total and specific IgE production [13, 346], but they are also protective from a positive SPT [346]. In summary, birth cohorts for studying the natural history of allergy in the Tropics are very few and just starting; however, they have provided important information about the influence of particular risk factors, mainly helminth and HDM exposure, on early sensitization.

### The role of mite exposure

Mite allergy is a worldwide problem and in the context of the Tropics it is probably the main cause of sensitization and symptoms. Although it is currently accepted that mite allergens cause respiratory and other symptoms, a lot of work remains to be done regarding the patterns of sensitization to purified allergens, their relationship with specific symptoms, and the immunogenetics of the IgE response. Why mite components are so allergenic is a fundamental unresolved question that belongs to the general theoretical problem of allergenicity. In the Tropics, this and other questions have to be analyzed in the context of perennial mite exposure, the immunomodulatory (suppression and/or enhancement) influence of helminth infections, an existent biodiversity, and probably specific human microbiota. This particular scenario should provoke the interest of the scientific community worldwide as it represents a potential source of new knowledge, as will be described in the following two sections.

### The high strength of mite specific IgE in the Tropics

HDM are the main risk factor for asthma in the Tropics [62]. Some of the effects of mite exposure on IgE sensitization and asthma have been described above; here and in the next section we present a more detailed analysis of the relationships between mite exposure and helminth infection on asthma. An interesting characteristic of allergy in the Tropics is the high rate of sensitization to HDM allergens and the high strength of the specific IgE response. This phenotype has been described in Asian as well as Latin American tropical countries. In some regions, perennial exposure may explain part of the hyperresponsiveness to HDM allergens, in others where helminthiases are still present, the cross reactivity between HDM and *Ascaris* (and other helminth) allergens also play a role [62, 354, 365, 366]. Furthermore, there is experimental and epidemiological evidence indicating that infection with the helminth *A. lumbricoides* induces a nonspecific boosting of the IgE response to mite allergens [367–369]. Genetic factors are less studied but in some populations with important African ancestry, genetic variants have been associated with high total IgE levels and asthma [347, 370–372]. In addition, the number of allergen sources sensitizing asthmatic patients in tropical zone seems to be low and restricted mainly to HDM [60]. The clinical impact of this hyperresponsiveness to mite allergens is partially supported by the great success of mite specific immunotherapy [373–378]; in addition, there is epidemiological evidence of relationship between sensitization and asthma and rhinitis [297] and also provocation tests using mite extracts [379–381].

### Tropical mite species with clinical impact

**Fig. 3:**
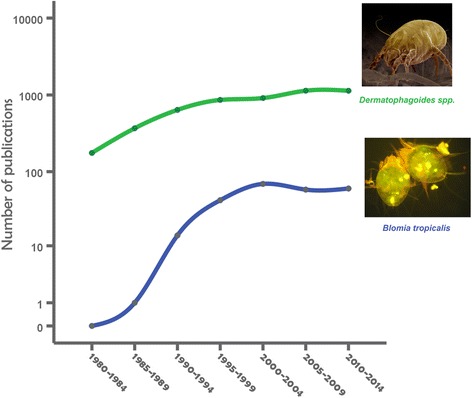
Research activities on HDM *B. tropicalis* and the genus *Dermatophagoides*, as detected by number of publications. Data source: Pubmed (until Jun 2014)

Numerous mite species have been described as the source of allergens capable of sensitizing and inducing allergic symptoms in genetically predisposed individuals. HDM are commonly present in human dwellings and are especially abundant in mattresses, sofas, carpets, and blankets. The most studied are *D. pteronyssinus*, *D. farinae* and *B. tropicalis* (Fig. 3). In addition, *E. maynei*, *D. microceras*, *D. siboney*, *Gymnoglyphus longior, Lepidoglyphus destructor*, *Glycyphagus domesticus*, *Tyrophagus putrescentiae*, *Acarus siro*, *Aleuroglyphus ovatus*, *Suidasia medanensis* and *Chortoglyphus arcuatus* have clinical relevance. Several allergens have been characterized in most of these species [382, 383]. Table 4 shows the species of mite that have been described as allergenic in populations from the Tropics. However, for some of them the IgE binding capacity by serological or skin test remains to be determined.

**Table 4:** Some house dust mite species from the tropics

Family	Genus	Species
Pyroglyphidae	*Dermatophagoides*	*pteronyssinus*
	*,,*	*farinae*
	*,,*	*sibonei*
	*,,*	*microceras*
	*Europhypus*	*mainei*
	*Hirstia*	*domicola*
	*Malayoglyphus*	*intermedius*
	*,,*	*carmelitus*
	*Pyroglyphus*	*africanus*
	*Sturnophagoides*	*brasiliensis*
Glycyphagidae	*Glycyphagus*	*domesticus*
	*,,*	*privatus*
	*Gohieria*	*fusca*
	*Lepidopglypus*	*destructor*
	*Astroglycyphagus*	*malaysiensis*
Echimyopodidae	*Blomia*	*tropicalis*
		*kulagini*
		*tjibodas*
Chortoglyphidae	*Chortoglyphus*	*arcuatus*
Acaridae	*Suidasia*	*medanensis*
	*,,*	*nesbitti*
	*,,*	*pontficiae*
	*Tyrophagus*	*putrescentiae*
	*,,*	*longior*
	*Acarus*	*siro*
	*Aleuroglyphus*	*ovatus*
Cheyletidae	*Cheyletus*	*eruditus*
	*,,*	*tenuipilis*
	*,,*	*malaccensis*
	*,,*	*fortis*
	*,,*	*trouessari*
	*Cheletonella*	*caucasica*
	*,,*	*vestertilionis*
Tarsonemidae	*Tarsonemus spp.*	

#### Prevalence and abundance

Latin America is one of the tropical regions with higher prevalence of *B. tropicalis* and *D. pteronyssinus*. Thus, these two species are the more common in Lima (Peru), Juiz de Fora (Brazil) [384–386], Cartagena and Santa Marta (Colombia) [386, 387] and Caracas (Venezuela) [388], with preponderance of *B. tropicalis* in some cities. In Central America, same species are very prevalent; in Puerto Rico *D. pteronyssinus* and *B. tropicalis* are the most prevalent followed by *D. farinae* and *D. siboney* [389]. In San José de Costa Rica, *B. tropicalis* is the most frequent followed by *C. arcuatus* and *D. pteronyssinus* [390]. In Cuba the more frequent are *D. pteronyssinus*, *B. tropicalis* and *D. siboney* [391]. Other species with low abundance in house dust samples are *L. destructor*, *S. medanensis* and *Cheyletus spp.* [392]. Several species of *Blomia* have been described, however *B. tropicalis* has the greater occurrence and sensitization to other species seem to be due to cross-reactivity [393, 394]. The mite fauna of house dust from tropical regions of India, Taiwan and Singapore is also dominated by *D. pteronyssinus* and *B. tropicalis* [260, 395], where important levels of their allergens have been quantified in homes from allergic patients. In contrast to temperate climates where marked fluctuations of HDM allergen levels have been reported, the tropical regions have few fluctuations because temperature and relative humidity is almost the same the whole year [238].

#### Clinical impact

The frequency of sensitization to *B. tropicalis*, *D. pteronyssinus* and *D. farinae* in asthmatics patients from different cities of Latin America have been reported between 60 and 97 %. Sensitization to mites of low abundance such as *C. arcuatus*, *A. ovatus* and *L. destructor* ranges from 21 to 71 % [257, 396, 397]. Although *D. farinae* is found in less abundance than *B. tropicalis* and *D. pteronyssinus*, sensitization to its allergens is similar to or greater than those for *D. pteronyssinus* or *B. tropicalis. D. siboney* is abundant in Cuba, where it has an important role as inducer of allergic diseases. Frequencies of sensitization higher than 80 % by skin test and higher than 95 % by RAST were initially informed by Ferrandiz R et al. [231]; recently, in a study based on skin test on 210 allergic patients, rates of sensitization were 78 % for *D. pteronyssinus*, 51 % for *D. siboney* and 56 % for *B. tropicalis* [398]. The presence of *D. siboney* is also reported in house dust samples from Puerto Rico [389] and Colombia [386]. *D. siboney* have been found to cohabit with *B. tropicalis*, *D. pteronyssinus* and *D. farina*e in the tropical rain forest region of China, where dust mite densities do not show significant variations across seasons [399].

The frequency of sensitization to *Suidasia medanensis* was 73 % in asthmatic allergic from Cartagena Colombia, in a study where an important degree of cross-reactivity between this species and *B. tropicalis* was found [400]. The genus *Suidasia* has been associated with anaphylaxis by contaminated foods in Venezuela. Using provocation tests Stanaland et al. [379] demonstrated that 83 % of *B. tropicalis* sensitive patients in Florida (USA) had a positive nasal challenge with *B. tropicalis* extract, indicating that skin test is a good indicator of allergic symptoms after inhalation of this allergen. In Brazil, 90 % of a group of patients had positive nasal challenge to *D. pteronyssinus* and 60 % to *B. tropicalis* [380]. In addition, patients who were sensitized to *D. pteronyssinus* and *B. tropicalis* might only react to one of them [381]. In a study that included 2206 children living in rural Taiwan a higher frequency of sensitization to *B. tropicalis* than to *D. pteronyssinus* was found, suggesting an important role of *B. tropicalis* in wheezing children [401]. Studies in Cartagena, Colombia and Florida, USA, showed that sensitization to these species was associated with acute asthma both in adults [297] and children [402]. Several investigators have informed about the importance of using *B. tropicalis* extract for a better diagnosis of mite allergy and also recommend to include it for an effective immunotherapy [250, 403]. Considering the great variety of mite species found in tropical house dust, it is possible that other, minor sources of allergens could be clinically relevant. For example, the IgE reactivity to *C. eruditus* and *C. malascencis* have been documented [382]. Also, frequency of sensitization to *Sturnophagoides brasilensis* and *Astroglycyphagus malaysiensis* has been reported between 50 and 70 % in allergic patients from Singapore [225].

#### Cross-reactivity among mites

The IgE cross-reactivity between *B. tropicalis* and *Dermatophagoides* is low to moderate [250, 404]. For allergens such as Blo t 1 and Der p1, absence of cross-reactivity has been documented [405]. Some allergic populations show a species specific IgE response toward Blo t 5 allergen, and the cross-reactivity between group 5 allergens has been found low to moderate when using whole recombinant allergens [251, 406]. This suggests that highly specific clinical reagents are necessary for precise diagnosis and immunotherapeutic treatment of sensitization to Group 5 mite allergens. The presence of species specific IgE components in *B. tropicalis* is illustrated by the study of 60 Taiwanese patients, where two major allergenic components of *B. tropicalis*, of about 14.3 and 27.3 kDa, were not inhibited by *D. pteronyssinus* [407]. Employing ELISA inhibition with sera from Colombian allergic patients 82 % of IgE cross-reactivity between *L. destructor* and *B. tropicalis* extracts was demonstrated [404]. An important degree of cross-reactivity was found by inhibition of immunoblot using sera from Brazilian allergic patients, this study suggested that Blo t 2 was the allergen responsible for the cross-reactivity, since it is cross-reactive with the recombinant Lep d 2 of *L. destructor* [408]. RAST inhibition studies using sera from asthmatic patients showed high degree of cross-reactivity between *B. tropicalis* and *S. medanensis* and suggested that *B. tropicalis* was the main sensitizer [400].

### The role of helminth infections

Helminthiases can influence allergic diseases by stimulating or suppressing the immune response, depending on the severity of the infection, which in turn is determined by host genetic susceptibility and the level of exposure. A number of epidemiologic surveys suggest that nowadays severe, chronic infections, with heavy worm loads and polyparasitism are still present in rural areas of the Tropics while intermittent and low intensity infections occur mainly in urbanized areas, with *A. lumbricoides* and *T. trichiura* being the most common parasites. As a consequence, helminthiasis is included among the risk or protective factors for allergy in the Tropics. During the last years it has become clear that ascariasis may influence several aspects of allergy, such as prevalence, diagnosis, severity and prevention. Since an inverse prevalence rate between helminthiases and allergy has been found it could provide insights into the reasons of the high prevalence of allergy in urbanized zones of the Tropics.

Theoretically all human infecting helminths can modify type 2 immune responses including the allergic reactivity but this has been more investigated in *Schistosoma, Ascaris*, *Filariae* and *Necator* species. The putative mechanisms underlying the connection between helminthiases and increased allergic reactivity involve: (1) induction of specific IgE antibodies against cross-reactive allergens; (2) helminth-induced boosting of specific IgE synthesis to bystander molecules; (3) maintenance of selected B and T cell clones by repeated infections and (4) perennial allergen exposure. In the next two sections we describe some of the most studied IgE binding molecules from helminths as well as several components with immunomodulatory properties, and discuss their potential effects on the allergic responses of individuals living in the Tropics. It is important to note that the IgE binding property is not sufficient for being a clinically relevant allergen and most of the IgE responses to helminth are related to parasite immunity and not necessarily to allergic reactions. In the following four sections we analyze the several topics of helminthiases with demonstrated or potential capacity to influencing allergic conditions in the Tropics.

### Helminth IgE binding components and their potential role in allergy

#### Ascaris

*A. lumbricoides* is the most common soil-transmitted nematode and compared to other helminth species is very allergenic. Several epidemiological surveys have found that ascariasis is a risk factor for asthma and atopy and that IgE response to *Ascaris* allergens is more frequent and stronger in mite-sensitized allergic patients. A number of IgE binding components in the *Ascaris* extract have been detected by human sera [365], but only three allergens are officially accepted by the WHO/IUIS Allergen Nomenclature Sub-Committee: tropomyosin (Asc l 3), glutathione transferase (GST, Asc l 13) and the nematode polyprotein ABA-1 (Asc s 1). Tropomyosin, a well-recognized pan-allergen, is involved in *Ascaris*-HDM cross-reactivity, which is explained by the high degree of structural homology between Asc l 3 and HDM tropomyosins [365, 409]. Specific IgE levels to Asc l 3 are significantly higher in asthmatic patients compared to healthy controls and suggest that it may be a risk factor for asthma symptoms in the Tropics [62]. The cross reactivity between *Ascaris* and HDM tropomyosins is one of the mechanisms explaining the enhanced IgE responses to tropomyosins in asthmatic patients living in the Tropics (reviewed in [410]). It has been proposed that patients predisposed to asthma, with a strong pro-Th2 genetic background, early age parasited, suffering several re-infections and permanently exposed to mite allergens probably have a stronger IgE responses to allergens and more severe clinical symptoms [411, 56]. The pseudocelomic fluid of *Ascaris* contains abundant amounts of the nematode polyprotein ABA-1 (Asc s 1). In humans, the IgE and IgG responses to ABA-1 has been associated with protection [412] rather than allergy symptoms, and no association has been found between IgE sensitization to ABA-1 and asthma. Indeed, ABA-1 is a nematode specific antigen, although present in other nematodes, it is not cross-reactive with any of the *B. tropicalis* or *D. pteronyssinus* allergens and is considered a nematode specific infection marker [365]. The frequency of IgE reactivity to Asc l 13 is low (<20 %) but these molecules exists as isoforms and may be clinically relevant in cases where there is also sensitization to mite GSTs [413]. The allergenic cross reactivity between *Ascaris* and HDM GSTs is suggested by the structural homology between Asc l 13 and allergenic GSTs in cockroach (Bla g 5) and HDM (Der p 8 and Blo t 8) however more studies are needed to prove this. The additional discovery of allergenic properties of Asc l 13 may explain why the frequency of IgE sensitization to invertebrate GSTs is higher in the Tropics than in populations living in temperate areas [414].

#### Filariae

IgE binding components have been described in *Brugia malayi* and *Onchocerca volvulus*. A homologue of gamma-glutamyl transpeptidase is a potent allergen of *B. malayi*, involved in tropical pulmonary eosinophilia (TPE) and the induction of antibodies against the pulmonary epithelium. IgE levels to this molecule are significantly elevated in patients with TPE and possibly induce resistance to the infection [415]. Tissue-invasive filarial infection increased the serological prevalence of IgE against HDM and cockroach, but not against timothy grass [366], probably due to the presence of tropomyosins [416]. Filarial GSTs are also recognized by IgE of infected patients; and comparative molecular studies between cockroach GST (Bla g 5) and *W. bancrofti* GST revealed that filarial infected subjects show IgE reactivity to both molecules [417]. Other filarial IgE binding components include the aspartic proteinase inhibitor Bm33 [418] and a fatty acid binding protein (BmAl-1) that seems to be specific and conserved in filariids and potentially allergenic [419]. The allergenic potential of filarial cystatin has been evaluated in a murine model but further studies in humans are needed [420].

#### Schistosoma

A group of proteins with orthologues in bee venoms, and thereby called Venom-Allergen-Like (VALs) are secreted during *Schistosoma* lifecycle [421]; among them SmVAL from *S. mansoni* is a strong inducer of human IgE. Some SmVALs are being proposed as vaccine candidates; however, their allergenic effects remain to be further explored [422]. Studies in mice models revealed that challenge with SmVAL4 increases the number of cells in the bronchoalveolar fluid, mainly eosinophils, induces dense peribronchiolar infiltrate, increases specific IgE levels and positive responses as detected by passive cutaneous anaphylaxis [423]. Other *Schistosoma spp.* IgE binding components are the tegumental allergen-like (TAL) proteins. Sm22.6 (SmTAL1) as well as Sj22.6 and Sh22.6 belong to this group. All induce IgE in infected humans and the IgE response to TAL1 is associated with resistance to re-infections [424–426].

#### Necator

There are two major *N. americanus* components that induce specific IgE antibodies: NaASP2 and NaCalreticulin. The first shares structural homology with the family PR1 (Pathogenesis related Proteins 1). High levels of IgE to NaASP2 is associated with low infection burden in human populations living in endemic regions [427]. Clinical trials evaluating vaccine candidates for *N. americanus* revealed that NaASP2 induced IgG_4_ antibodies [428] but in some individuals triggered generalized urticaria. Mean IgE levels to NaASP2 were 21 times higher in subjects with urticaria compared to those without symptoms [429]. Nacal1 binds IgE in all subjects with detectable antibodies to the *Necator* extract [430] and induced basophil degranulation and histamine release in infected subjects from Madang, New Guinea [431].

#### Toxocara

Human specific IgE antibodies detect several components in the excretory-secretory products of *T. canis* [432]. The infection with *Toxocara* exacerbates allergic reactivity to ovoalbumin in a murine model, and increases expression of IL-4 in the lungs [433]; in addition, several epidemiological studies have shown positive association between seropositivity to *Toxocara* and asthma. Although toxocariasis occurs worldwide, it is more frequent in developing tropical regions and its effects on allergen reactivity vary depending on the context of the infection. In some areas toxocariasis is associated with increased markers of Th2 immune response and IgE sensitization to aeroallergens but also with suppression of hypersensitivity reactions in skin [434, 435].

#### Anisakis

Allergic symptoms are the most known effects of *anisakiasis*. *Anisakis typica* is common in the Tropics and *Anisakis simplex* in the northern hemisphere. Officially recognized allergens are Ani s 1 to Ani s 14, being Ani s 1 the major allergen [436]. This molecule induces hypersensitivity reactions in skin tests and basophil degranulation in individuals sensitized to *Anisakis* [437]. Ani s 2 is the *Anisakis* paramyosin and is recognized by specific IgE antibodies in 88 % of individuals sensitized to this nematode [438]; together with Ani s 3 is considered a helminth pan-allergen [439]. Ani s 3 is the *Anisakis* tropomyosin, which has IgE cross reactivity with tropomyosin from HDM, cockroach and shrimp [440], although it is not an important sensitizers among patients [437]. More studies are needed to define the role of these and other *Anisakis* allergens on the IgE responses to allergens from non-parasitic sources.

In summary, depending on the presence of polyparasitism, the overlap in the antigenic composition between HDM, *Ascaris* and other nematodes could elicit diverse immune responses and modify the effects of helminth allergens on allergic diseases in the Tropics.

### Helminth immunomodulators influencing allergy symptoms

There is increasing interest about the immunomodulation associated with chronic helminth infections, which in some rural areas make the population more susceptible to viral infections and jeopardize vaccination programs. The idea that parasite infections protect from allergies is mainly derived from the indirect observation that, in endemic populations, asthma prevalence is lower than in high-income countries where parasites have been almost eradicated [441]. Numerous contradictory epidemiologic results have been published around this topic, but basic research has shown that, independently of the effects at the population level, helminths have immunomodulatory components or induce host derived immunomodulatory pathways that can influence the evolution of allergic diseases. In vitro experiments evaluating the effects of helminth on infected hosts point out a clear suppressive effect of several helminth species on the immune response. Larva, egg or somatic worm extracts as well as purified natural or recombinant products have proven to be excellent stimuli for IL-10 or TGF-beta production, both anti-inflammatory cytokines. In murine models of asthma or airway hypersensitivity, helminths or their products induce different mechanisms that may ameliorate the allergic phenotype [442, 443]. There have been also controversial clinical trials in humans suggesting that helminth infections (used as treatment) can control allergy symptoms, and the potential of this strategy has been critically analyzed [444]. A list of helminth immunomodulators from different species is described in Table 5.

**Table 5:** Some helminth immunomodulatory products

Name	Helminth	Effects
Cystatin	*A. vitae*	Inhibition of antigenic processing. Suppression of Th2 inflammation in airway hypersensitivity models and Th1 driven responses in experimental colitis. Induction of IL-10 production.
ES-62	*A. vitae*	Decreases inflammation in rheumatoid arthritis model through IL-17 blockage. Inhibition of mast cell activation and allergic reactions in airway sensitization models.
TGF-beta like factor	*H. polygirus, B. malayi*	Inhibition of airway inflammation, possibly through binding to mammalian TGF-beta receptor.
SM-16	*Schistosoma* spp.	Suppression of thioglycollate-induced peritoneal macrophage maturation. Prevention of classical macrophage activation.
Galectin	*T. leonine*	Reduction of clinical manifestations of experimental colitis. Th1 and Th2 inhibition by increasing IL-10 and TGF-B production.
PAS-1	*A. suum*	Reduction of allergic responses in airway hypersensitivity models. It also increases IL-10 and TGF-beta production

Several studies have shown that helminth infections expand the number of regulatory T cells (Tregs) [445, 446]. Besides, natural thymus-derived Tregs, other circulating population secreting IL-10 or TGF-β, have been identified [446, 447]. Helminth induced Tregs have immunosupressive properties on other T lymphocytes [448] through cytokine secretion or cell-cell contact mechanisms (by means of CTLA-4 and GITR) [449, 450]. Tregs can induce specific hyporesponsiveness to helminth antigens but also to bystander allergens [451]. Helminth infections can also induce regulatory B cells (Bregs) [452]. In mice, it was shown that the suppressive activity of *S. mansoni* extracts on airway allergic inflammation was dependent on IL-10 producing B cells, mostly found in the spleen marginal zone. These cells were found to promote conversion of T cells to a regulatory CD25^+^ Foxp3^+^ phenotype. Other cells are also subject of immunosuppression, for example, chronic ascariasis decreases basophil activation [453]. Induction of high levels of total IgE and IgG_4_ by helminths may diminish the effector mechanisms of allergy, such as histamine release by mast cells and basophils. In general, more research is needed to define the extent of helminth-induced immunomodulation and its effects on the allergic responses during or after natural human infection, which is known to be also associated to helminth-induced immunostimulation.

### Intestinal microbiota and parasites

In recent years it has become clear that the human microbiota, in its different locations, plays an essential role in the development and function of the immune and other systems. In the Tropics, a particular relationship emerges with potential repercussions on the general physiology of humans which is the co-existence of bacterial commensals and parasites in the intestine. The composition of human intestinal microbiota is determined by several factors, most of them undefined. It is known that competition for microbial nutrients, the immunomodulator properties of some bacteria, the diet and the host innate immune responses might play a role; however the effects of helminth infections on microbiota composition and the role of microbiota in helminth immunity and its effects on allergy have been poorly analyzed. A brief comment on this topic is very pertinent because the co-evolutionary history of digestive system, immune system, microbiota and helminth infections anticipates tight links in between. Research on these topics has provided interesting data that could lead to new interrogates regarding the effects of helminthiases on the pathogenesis of allergic diseases. First, several studies have found that alterations of the microbiota promote Th2 responses, specific IgE to mite allergens, asthma susceptibility and asthma severity [454, 455]. Second, there is evidence that helminth infections increase asthma susceptibility [456] and severity [232], Third, helminth infections can alter the microbiota composition but the consequences on its immunomodulatory effects have not been evaluated. Therefore, it is possible that alterations of human intestinal microbiota by helminth infections are an additional contribution to the pro-allergic effects of helminthiases in the Tropics.

### Genetics of helminthiases and allergy in the Tropics

Genetics of complex traits such as allergy and immunity to helminths are matters of study worldwide, but it acquires special interest in the Tropics because of the great number of shared aspects between the allergic reaction and the immune response to helminths. There is no unique genetic background for people living in the Tropics. Depending on their historical past, countries in this zone are the results of diverse population admixtures from most of the current world populations. African ancestry is an important component of the genetic admixture in several tropical regions and some studies suggest that this component predisposes to high levels of total IgE and asthma, which could be the only known risk at the population level; however, there are not enough data supporting that this ancestry component exerts influence in all genetic backgrounds. An advantageous condition for studying the genetics of IgE responses in the Tropics is that extreme phenotypes such as IgE hyperresponsiveness to allergens and helminth components allow a better dissection of the genetic and environmental influences. Epidemiological studies revealed that in addition to exposure from the household, genetic factors account for an important proportion of the variation in worm loads [457–460]. A summary of genes associated with the susceptibility to helminth infections in humans [461–474] is presented in Table 6.

**Table 6:** Genetic variants associated with the immune response to helminths

Gene	Locus	Product	Variant	Effect	Helminth	Population
*IL10*	1q31-q32	Interleukin 10	rs3024492	Allele C of rs3024498 was negatively associated with helminth infection	*Ascaris lumbricoides*	Brazil
*CHIT1*	1q32.1	Chitinase 1 chitotriosidase	H allele: 24 bp duplication in exon 10	Homozygotes HH low levels of chitotriosidase. H allele over-represented in the infected group	*Wuchereria bancrofti*	South India
-	2p21-p14	Unknown	D2S2378	Linkage between the numbers of microfilariae and markers at 2p21-p14	*Onchocerca volvulus*	Ghana
*TLR2*	4q32	Toll receptor 2	−196 to −173 del	Higher risk of asymptomatic filariasis	*Wuchereria bancrofti*	Thailand
			+597 T > C			
			+1350 T > C			
*IL13*	5q31	Interleukin 13	Arg110Gln	Gln/Gln: Strong Th2 reactivity, low microfilarial number	*Onchocerca volvulus*	Guinea
			−1055 C > T	Less *Ascaris* burden	*Ascaris lumbricoides*	China
			rs7719175	Haplotypes rs7719175T and rs1800925C associated with high infection levels	*Schistosoma haematobium*	Mali
			rs1800925			
*MBL2*	10q11	Mannose-binding lectin 2	Low –expressing MBL	Increased prevalence of positive circulating filarial antigen (CFA status)	*Wuchereria bancrofti*	Tanzania
			Defective C allele	Three times more probability to be infected with Filariae		
*STAT6*	12q13	Signal transducer and activator of transcription 6	Microsatellite in exon 1	Carriers of the short allele plus SNPs haplotypes have lower levels of *Ascaris* infection	*Ascaris lumbricoides*	China
			5′UTR/3′UTR haplotypes			
			3′UTR SNP 4219 G/A	Homozygotes GG have the lowest egg counts	*Ascaris lumbricoides*	China
-	13q33-34	Unknown	D13S1265 D13S285	Linkage with egg counts of *A. lumbricoides* in stool and total IgE levels	*Ascaris lumbricoides*	Nepal
*TNFSF13B*	13q33	B Cell activating factor	G3980 > C	GG homozygotes have higher levels of IgG to Ascaris	*Ascaris lumbricoides*	Colombia
*TGFB1*	19q13.1	Transforming growth factor beta 1	Leu10Pro	Leu/Leu: Latent infection	*Wuchereria bancrofti*	Ghana

#### Common genes for helminth responses and allergic phenotypes

Considering the overlap in biological pathways implicated in the immune responses to helminths and allergens, it has been hypothesized that some genetic variants influencing the IgE response to helminths may also predispose to IgE sensitization with non-parasitic allergens [470] or even predispose to allergic diseases [475, 476]. Peisong et al., found an association between an asthma-related genetic variant of *STAT6* and *Ascaris* egg counts in China [466, 470]. The 5q31 locus is an example of a common locus for the susceptibility to helminthic infection and allergic diseases, as shown by the linkage with the susceptibility to *S. mansoni* and the genetic associations between *IL13* polymorphisms with the immune response to *O. volvulus*, implicating a coding variant (Arg110Gln) that has been previously associated with asthma and other allergies [477, 478]. Genetic variants in the gene encoding chitotriosidase (*CHIT1*) have not only been associated with the response to filarial infection but also with asthma [479, 480]. Shared genetic variants between immune responses to helminth and susceptibility to allergic diseases are not common considering the great number of variants found associated with asthma and other allergic diseases. Few studies have evaluated simultaneously the effects of genetic variants on the susceptibility to helminthiases and asthma in the same population. Following this approach it was found that variants of the gene *TNFSF13B* encoding for the cytokine B Cell Activating Factor (BAFF) and other genes in the 13q33 locus were associated with the IgE response to *Ascaris* but not the IgE response to HDM allergens [473]. The tropical scenario, where helminth infections and allergies are very frequent, is ideal for exploring the genetic and evolutionary roots of both conditions.

### Particular aspects of allergy diagnosis and management in the Tropics

As expected, tropical conditions also affect diagnostic criteria and management. Some of them are easy to understand; for example, the predictive value of total IgE might be limited by the polyclonal expansion of IgE producing B lymphocytes induced by helminth infections. But others, such as reagents for component resolved diagnosis (CRD) or mite allergens avoidance require more studies considering the great variety and level of exposure to mite allergens. In the following five sections we will discuss several circumstances where conventional diagnostic tools and management have limitations or particularities that should be considered in clinical practice and future research in the Tropics.

### Usefulness of total IgE and other diagnostic tests

It is well known that patients with allergies, especially asthma, have higher serum IgE levels compared to non-allergic subjects [481, 482] but abnormally higher IgE levels have also been described in a variety of diseases such as helminthiases, primary and secondary immunodeficiencies, graft versus host disease, chronic infections and neoplasms. Serum IgE levels, peripheral blood eosinophilia and skin test reactivity to allergens have been used to identify atopic individuals. The diagnostic value of allergen specific IgE is accepted but the predictive value of total serum IgE levels is controversial anywhere. Helminthiases are frequent inducers of high serum total IgE [483], therefore this test is not useful for diagnosing asthma or other allergic disease in individual cases. Because of its non-specific nature [484, 485], total IgE has been replaced by the specific IgE to allergens as a good marker of both, atopy and allergic diseases. However, there is an additional problem in the Tropics which is the impact of cross-reactivity in the diagnosis of mite allergy, the most important risk factor for asthma. *Ascaris* cross-reactive allergens, such as tropomyosin and GST, may influence mite allergy diagnosis when using the whole mite extracts, as is routinely done in vitro and for skin testing. Therefore, in the Tropics the use of complete mite extracts for diagnosis could lead to false positive results. Component resolved diagnosis, including mite and *Ascaris* purified allergens, could help to resolve this problem.

Another limitation of serologic allergy diagnosis in patients with helminthiases is the presence of the carbohydrate epitope galactose-α1,3-galactose (α-Gal), expressed in non-primate mammalian proteins, such as the cat allergen Fel d 5 (cat IgA). It has been reported that the diagnosis of cat allergy may be impaired among parasited patients [344]. There are other reports suggesting that intestinal helminths have α-gal [486], but immunochemical studies on *Ascaris* extract show that deglycosylation treatment does not significantly affect the specific IgE antibody binding to the extract [365]. Further research is needed to confirm the presence of this cross-reactive carbohydrate in *Ascaris*. It has been reported that skin test reactivity to clinically relevant allergens is low among children with chronic severe helminthiases, including ascariasis [53], although the IgE response to allergens can be demonstrated in those patients using serological methods. This epidemiologic finding may be not visible at the individual patient-physician level; however, if sensitization to allergens is evaluated in these communities using skin tests, false negative results will be common, and this may enforce the opinion that not only allergy symptoms but also allergen sensitization are infrequent in parasited populations. Allergists in the Tropics have to follow different criteria for diagnosing allergic diseases and other diseases known to have increased IgE production. Total serum IgE determination for assessing the suitability of a patient for anti-IgE treatment may be difficult in clinical settings [487].

### Component resolved diagnosis for mite allergy

Several studies have intended to define the minimum number of components needed to replace mite extracts. This has been achieved for *D. pteronyssinus*, with potential clinical usefulness in temperate and tropical countries. However, in the case of *B. tropicalis* more research, including new recombinant allergens, is required to replace the whole extract. In addition, a particular point in the Tropics is the abundance and diversity of mites leading to frequent and strong sensitizations to different species due to both, co-sensitization and cross reactivity. This makes difficult the selection of extracts for immunotherapy, a problem that could be resolved if species specific components were found. Currently there is no platform designed for CRD in the Tropics, where more than 80 % of allergic patients are sensitized to any of the HDM species *B. tropicalis* or *D. pteronyssinus* [396]. Around 10 % of allergic patients are mono-sensitized to *B. tropicalis* and a similar proportion is detected for *D. pteronyssinus* by current diagnostic methods [488], even though people are co-exposed to both species [489]. Most of allergic patients, double-sensitized, receive immunotherapy with an admixture of the two sources. It is unknown in which extent one of the two preparations is unnecessarily administered since cross-reactive components are frequent sensitizers. Another potential problem is the bias in diagnosis that causes IgE cross-reactivity between HDM and helminth antigens that frequently induces specific IgE production in the exposed population [365, 490]. Therefore, IgE testing of species-specific markers as well as cross-reactive molecules could help to define the most appropriate reagents for mite allergy immunotherapy in the Tropics.

Some attempts have been made to replace the HDM extracts using recombinant components in the Tropics. In Colombia, the combination of Der p 1, Der p 2 and Der p 10 detected 93.5 % of the *D. pteronyssinus* positive sera [491]. In Brazil, Blo t 5 and Blo t 21 detects 96.4 % of the responders to the whole body extract [255]. In Singapore, by testing 14 components from *B. tropicalis*, Kidon et al. found that the combination of Blo t 5, Blo t 21 and Blo t 7 detected about 70 % of SPT positive patients. In the case of *D. pteronyssinus* among 14 allergens, Der p 1 and Der p 2 identified approximately 78 % of *D. pteronyssinus* extract responders. Inclusion of other allergens did not increase the sensitivity of the battery. Regarding the combination of allergens for selecting the appropriate extract (*B. tropicalis* vs. *D. pteronyssinus*) for immunotherapy, it can be hypothesized that using Der p 1, Der p 2, Blo t 5 and Blo t 12 in different steps of the diagnostic process could be a good approach.

### Component resolved diagnosis for pollen and mold allergy

Pollen sensitization profiles vary in different tropical countries and are certainly different to those found in more temperate regions. Regarding grass species, the subtropical/tropical grasses of the *Chloridoideae* (e.g. *Cynodon dactylon*; Bermuda grass) and *Panicoideae* (e.g. *Paspalum notatum;* Bahia grass) species are abundant in parts of Africa, India, Asia, Australia and the Americas [281]. Patients sensitized to these species show partial cross-inhibition of IgE reactivity by temperate grass pollen allergens indicating the presence of species-specific IgE binding sites in subtropical grass pollen allergens that are not represented in temperate grass pollens. This fact has important implications when designing allergen-specific immunotherapy to subtropical grass pollen allergens [492]. A study performed in Zimbabwe, central Africa, revealed that compared to central European grass pollen allergic patients, African patients did not recognize Phl p1, Phl p 5 or Phl p 2 as major allergens, but a higher recognition of Phl p4 was detected, possibly by a predominant sensitization to Bermuda grass which preferentially expresses species-specific allergens rather than cross-reacting molecules. Specific IgE to profilin and to Bet v 2 was extremely prevalent, while Phl p 12 was much lower [493]. A recent study has revealed an astonishing high frequency of IgE sensitization to non-allergenic, grass pollen–derived carbohydrate epitopes and the absence of IgE reactivity to highly allergenic grass pollen protein allergens in the Philippines, indicating that the local flora consisting of tropical grasses does not induce clinically relevant sensitizations [494].

A recent publication assessed the usefulness of a multiplex allergen microarray in atopic patients from Singapore. The authors suggest that this tool is not optimal for general screening, especially in a population with low rates of polysensitization and a dominant sensitizing allergen (HDM), but propose that it may have a role if the allergen molecule panel is adjusted to regional differences [495]. Another relevant source of allergens in the Tropics are molds, and indeed, allergic patients frequently display mold sensitization [63, 496]. Nevertheless, component resolved diagnosis in fungal allergy has been mainly limited to *Alternaria* and *Aspergillus* species, and knowledge in this area is still scarce [497]. These data suggest that indeed there is a role for CRD in order to define true sensitization to a specific pollen allergenic source and to evaluate the role of certain panallergens in the Tropics.

### IgG/IgE ratio and allergy symptoms

IgG/IgE ratio is emerging as an important parameter in clinical allergy. First it was noticed that the IgE responses to diverse allergen sources was accompanied with IgG antibodies to the same allergens [498]. Normal population had mainly IgG while allergic subjects showed predominantly IgE [499], suggesting an unknown physiologic role for IgG, in relation to a non Th2 response. An important step to understanding the role of IgG responses, especially IgG_4_, has been the analysis of allergen specific immunotherapy mechanisms. Several studies have shown that SIT-induced IgG_4_ can act as blocking antibodies [500]. Furthermore, the potential allergy-protective role of IgG4 antibodies has been identified in food sensitized but clinically tolerant individuals [501].

In the Tropics most research on this topic has been performed around parasitic infections; however some preliminary data on food allergy is available [502]. Despite this, to date the exact value of measuring the IgG/IgE ratio to predict the clinical response to a specific allergen remains unclear and it is, therefore, not currently recommended as a diagnostic tool [503]. A study in Brazil has shown that *D. pteronyssinus* specific IgG_4_ is associated with reduced levels of IgE to this mite, resulting in 35–51 % inhibition of IgE reactivity in atopic patients, but no clinically relevant data were reported [504]. Also, in Brazilian patients with AD, *B. tropicalis*-specific IgG_4_ was probably induced by chronic exposure to this mite in the home environment [505]. In cat allergy, studies have shown that high levels of IgG antibodies also provide a further marker of cat allergen exposure among Japanese patients with asthma [506]. Taken together, these data support the assumption that IgG antibodies are markers of exposure, and they do not allow inferring clinical reactivity in tropical areas. However, it has been found that chronic infections such as schistosomiasis and chronic exposure to laboratory animals promote IgG_4_ antibodies that could prevent allergic symptoms induced by the concurrent IgE-response [507, 508]. Therefore, it is possible that parasite infections could modify a parameter of potential clinical usefulness that is IgG_4_/IgE ratio. As in other places of the world, this aspect should be further investigated in the Tropics.

### The effectiveness of allergen quantification and avoidance

Allergen avoidance is a general recommendation in clinical practice for reducing frequency of symptoms; even though its effectiveness is not well documented. Also, it is still an open question if HDM allergen avoidance is a good primary prevention strategy since there is some evidence, mostly derived from temperate countries, about a dose–response relationship between HDM exposure and allergen sensitization. Systematic reviews about this topic, including studies from all over the world, have concluded that interventions to reduce HDM exposure do not significantly improve asthma control [509, 510]. It is of great importance to know how useful avoidance measures in the Tropics could be, considering that the climate predisposes to high HDM allergen levels in a constant manner (no seasonal variations). However, few studies have been done in tropical or sub-tropical countries, most of them with small sample size. From this limited data, positive results have been observed with some control measures.

In Egypt, 106 HDM allergic asthmatic children were allocated into one of four different intervention groups for HDM avoidance (chemical, physical, both chemical and physical or none). Physical measures included proper ventilation practice, completely bed covering and cleaning advice. Chemical interventions consisted of spraying carpets and bedding with tannic acid 3 % twice weekly. Sixteen weeks later, all interventions, especially the combination of both methods, reduced Der p 1 levels but not more than 20 % from baseline. The group for which physical control measures were used showed significant improvement in all outcomes. The control group (no intervention) also showed reduction of clinical symptoms, although this was more marked in physical intervention. Advantage of this study is that they used simple and not expensive measure such as HEPA filters [511]. Another study from Singapore evaluated bed covers and HEPA filters as two control methods to reduce HDM exposure in a small sample of asthmatic children (*n* = 24). Bed covers, but not HEPA filters, were effective for reducing Der p 1 and Der f 1 levels but not significantly associated with lung function improvement [512]. In addition, its efficacy lasted for only two months which means that they must be washed or replaced. In conclusion, although physical methods to reduce HDM exposure are partially effective, cleaning advice and bed covers seems to be recommendable to reduce allergic symptoms. Intervention studies to identify the relationship between allergen levels and development of allergic diseases have not been performed in tropical countries. It is possible that conventional cut-offs to define high level of exposure to HDM are not applicable to tropical regions in terms of incremental risk for allergy development.

### Ongoing changes in lifestyle

Lifestyle changes are suspected to influence the increasing frequency of allergic diseases worldwide [513]. Sedentary and indoor lifestyles, changes in the diet and hygiene improvement have repeatedly been associated with the increasing trend of allergy. More recently biodiversity loss and climate change [514, 515] have been added to the long list of potential explanations. In the Tropics the most significant environmental and lifestyle changes have been related to urbanization. In addition to the changes that urbanization brings everywhere, this process has implied security risks when occur in association with extreme poverty leading to changes in home design and increased time indoors. The impact of these changes on mite sensitization and respiratory allergy has not been evaluated.

### Unmet needs and perspectives

This review analyzed particularities of allergy in the Tropics, revealing important areas that require further investigation. Allergy training in tropical countries is out of the scope of the present review but will be analyzed in depth in a special document. Among the most important gaps is the role of pollens and molds as risk factors for asthma and rhinitis. This should be addressed in a collective effort to obtain comparable results and to test native allergens. Other topics that mandate further investigations include the prevalence and characteristics of stinging insects allergy; the prevalence and causes of Oral/Allergy syndrome; the relationship between helminthiases and urticaria; the epidemiology of drug hypersensitivity; the importance of tropomyosins in clinical practice of respiratory allergy; the clinical relevant allergens that should be included for CRD; the influence of helminthiases in the IgG/IgE ratio to common allergens; the prevalence of sensitization to alpha-gal and other carbohydrates and their diagnostic impact and the mechanisms underlying the effects of latitude and urbanization. Given the limitations of mite exposure avoidance in an environment where these organisms grow so easily, mite specific immunotherapy in the Tropics also requires further studies. Also, the search for helminth derived immunomodulators that could compensate for the effect of progressive decline of parasite infections should be stimulated, bearing in mind the potential adverse side effects on the immune responses to viral diseases. Genetic and epigenetic studies of atopy and allergy susceptibility could be more informative in the unique environmental conditions of the Tropics than in temperate climes. Since several research groups focused in both clinical and basic sciences are increasing in the new “New World”, the prospects for the field of allergy are promising.

## Conclusions

This review supports the idea that the field of allergy in the Tropics has particularities that deserve special attention from the scientific community and clinicians everywhere because they strongly affect the natural history of common allergic diseases. Defining epidemiological trends, risk factors and management possibilities will help numerous patients living in the tropical zone; therefore they should be part of specialty training programs in hospitals and universities. At the same time, this knowledge will help to better understand the pathogenesis and evolution of allergic diseases in temperate zones. We also noticed an emerging “allergy community” from several research centers in developing countries addressing fundamental and practical questions about the origin of allergy in tropical settings and raising interesting hypotheses whose solutions will involve both senior researchers as well as basic science and clinical fellows in training.

## Abbreviations

HDM, House dust mite; FRAAT, Risk Factors for Asthma And Atopy in the Tropics; AD, Atopic dermatitis; ISAAC, Study of Asthma and Allergies in Childhood; AU, Acute urticaria; CU, Chronic urticaria; PU, Papular urticaria; OMA, Oral mite anaphylaxis; FA, Food allergy; CB, Cord blood; TPE, Tropical pulmonary eosinophilia; CRD, Component resolved diagnosis

## Additional files

Additional file 1: Table S1. Regression analysis of ISAAC Centre characteristics and atopic conditions. (DOCX 14 kb) Additional file 2: Table S2. Overview of ISAAC centres by age and country, for wheeze and rhinoconjunctivitis. (XLS 122 kb) Additional file 3: Table S3. Allergen sensitization and asthma symptoms. (DOCX 25 kb) Additional file 4: Table S4. Food Sensitization in Children Living in the Tropics and Subtropics. (DOCX 30 kb) Additional file 5: Table S5. Overview of tropical studies examining pet sensitization in children. (DOCX 24 kb)
