# Supplementary material for: Particularities of allergy in the Tropics
Source: World Allergy Organ J. 2016 Jun 27;9:20. doi: 10.1186/s40413-016-0110-7 (PMC4924335; doi:10.1186/s40413-016-0110-7)
Supplement: Additional file 1: Table S1. — Regression analysis of ISAAC Centre characteristics and atopic conditions. (DOCX 14 kb) [file 40413_2016_110_MOESM1_ESM.docx]

| **Additional file 1: Table S1: Regression analysis of ISAAC Centre characteristics and atopic conditions** | | | | | | | | |
| --- | --- | --- | --- | --- | --- | --- | --- | --- |
| Outcome of interest | Altitude | | Latitude | | Longitude |  | Economic Status | |
|  | Beta-coefficient | p-value | Beta-coefficient | p-value | Beta-coefficient | p-value | Beta-coefficient | p-value |
| Current rhinitis: Whole Cohort |  |  |  |  |  |  | 0.380 | <0.001 |
| Current rhinitis: Age 13-14 years |  |  |  |  |  |  | 0.412 | 0.001 |
| Current rhinitis: Age 6-7 years |  |  |  |  | -0.391 | 0.007 | 0.500 | <0.001 |
| Hay Fever Ever: Whole Cohort |  |  | -0.360 | <0.001 |  |  |  |  |
| Hay Fever Ever: Age 13-14 years |  |  | -0.365 | 0.002 |  |  |  |  |
| Hay Fever Ever: Age 6-7 years | -0.367 | 0.034 |  |  |  |  |  |  |
| Change in prevalence between ISAAC Phase I and Phase III, of Hay Fever Ever: Whole cohort |  |  |  |  | -0.409 | 0.039 |  |  |
| Change in prevalence between ISAAC Phase I and Phase III, of Hay Fever Ever: Age 6-7 years | -0.597 | 0.038 |  |  | -0.860 | 0.009 |  |  |
| Current rhinoconjunctivitis: Whole Cohort |  |  | -0.196 | 0.022 | -0.299 | 0.002 | 0.257 | 0.004 |
| Current rhinoconjunctivitis: Age 13-14 years |  |  | -0.226 | 0.048 |  |  | 0.277 | 0.022 |
| Current rhinoconjunctivitis: Age 6-7 years |  |  |  |  | -0.560 | <0.001 | 0.477 | <0.001 |
| Current Wheeze: Whole Cohort | -0.281 | 0.001 | -0.230 | 0.005 | -0.485 | <0.001 |  |  |
| Current Wheeze: Age 13-14 years |  |  | -0.255 | 0.023 | -0.371 | 0.003 |  |  |
| Current Wheeze: Age 6-7 years | -0.574 | <0.001 |  |  | -0.716 | <0.001 |  |  |
| Asthma Ever: Whole Cohort | -0.383 | <0.001 | -0.201 | 0.011 | -0.345 | <0.001 | 0.318 | <0.001 |
| Asthma Ever: Age 13-14 years | -0.283 | 0.009 | -0.241 | 0.019 | -0.241 | 0.031 | 0.386 | <0.001 |
| Asthma Ever: Age 6-7 years | -0.578 | <0.001 |  |  | -0.518 | 0.001 | 0.277 | 0.033 |
