# Supplementary material for: Particularities of allergy in the Tropics
Source: World Allergy Organ J. 2016 Jun 27;9:20. doi: 10.1186/s40413-016-0110-7 (PMC4924335; doi:10.1186/s40413-016-0110-7)
Supplement: Additional file 3: Table S3. — Allergen sensitization and asthma symptoms. (DOCX 25 kb) [file 40413_2016_110_MOESM3_ESM.docx]

| Additional file 3: Table S3. Allergen sensitization and asthma symptoms | | | | |
| --- | --- | --- | --- | --- |
|  | Geographic Location  Study Design | | No. of subjects | Allergen/sensitization in Asthma/wheeze (unless otherwise stated) |
| **ASIA** |  |  |  |  |
| **HK/China** |  |  |  |  |
|  | HK/Guangzhou | ISAAC phase 2, Cross sectional population | 3110/3565 school children mean 10 yrs, SPT | HK Population Prevalence Dp 34%, Df 26%, cockroach 11.5%, Cat 3.7%  Association with current wheeze OR: Dp (odds ratio (OR)=4.48; cat (2.59), Df (2.41) |
|  | HK | Case control, Hospital clinic SPT and RAST | 176 cases mean 9.8yrs (SD3.7)and 57 age matched controls | SPT Dp 88%, Df 87%, Cockroach33%, Cat 40%, Dog 22%  Sensitization to HDM and cat were risk factors for asthma |
| **Taiwan** |  |  |  |  |
|  | Taipei,Taiwan | Clinic based  Immunocap and SPT | 131 asthma cases. children and adults | Dp 71% Df 69%, Bt 44%*, Per a* 37%, Bla g 36%, cat=8%, dog=4%, mosquito=29% |
| **Indonesia** |  |  |  |  |
|  | Jakarta Indonesia | Case controls SPT | 107, 32 children, 75 adults. 20 controls | Dp 78%, Bt 72%, *A. malaysiensis* 34%; *E. guineensis*(pollen-palm oil)*,* 22.3%, A*cacia. Auriculijormis* 12.15%; fern spore:*Dicranopteris* spp. 11%; fungal spore:*Curvularia fallax*(8 %) and *E. rostratum 13%.* |
| **Thailand** |  |  |  |  |
|  | Bangkok | Clinic based. Case controls SPT | 84 asthma children 2-14 years | Dp and Df 58%, Bt 10%, A malaysiensis 7%, Per a and Bla g 27%, *E. guineensis* (oil palm pollen) 8%, mold, spores <5%, |
|  | Patumthani (near Bangkok) | Allergy Clinic Case  SPT | 120 Children 3-15 yrs | *Dp 79%, Df* 69%, Per a 30%, Kapok 24%, Bla g 20%, Bermuda grass 16%, Johnson grass 15%, Cat 13%, Acacia 11%, Dog 8% |
|  | Songkla Province. South Thailand | Allergy clinic. Retrospective  SPT | 99 asthma children mean age 7 years | Dp 50.5% Df 52.3%, Per a 20.2%, cat 10%, dog and weeds and mold 3% |
| **Malaysia** |  |  |  |  |
|  | Kuala Lumpur | Cross sectional, Immunocap | 192 children, asthma and AR , 2-15 yrs | *Dp* 63% vs 23, Bt63 |
|  | Kuala Lumpur | Cross sectional, EIA | 49 asthma children | Blo t 5=90%, Der p 1=57%, Der p 2=39% |
| **China** |  |  |  |  |
|  | Guangdong(multicentre CARRAD) | Allergy centre cases  SPT | Total 6304, South China 2765 | Der p 88% , Per a 26% Bla g 19% |
|  | Guangdong | ISAAC phase 3. Cross section school children  SPT | 2531 children 13-14yr | Sensitization to Dp associated with wheeze |
| **Singapore** |  |  |  |  |
|  | Singapore | Allergy Clinic Cases  *In vitro* IgE | 253 asthma 119, 8 yrs | Asthma: Der p 91%, Bt 75%. All atopy: Der p 1=64%, Der p 2=71%, Blo t 5 =45%, Blo t 7= 44%, Blo t 21=56% |
|  | Singapore | Clinic cases Volunteer controls, SPT | 289 asthma and/or AR, 102 controls | Bt 96%, Dp 93%, Df 92%, A. malaysiensis 78%, S. brasiliensis 72%, T. putrescentiae 71%, canary feathers 70%), Per a 60%, Bla g 56%, mosquito (Aedes sp.) 46%, dog 34%, kapok seed 32%, cat 29%, Aspergillus fumigatus 21%), Penicillium notatum 18%, |
|  | Singapore | Cross section, clinic cases and volunteer controls  SPT – pollen, spores and mold | 231 subjects (asthma and /AR) and 76 controls | Oil-palm pollen (Elaeis guineensis) 40%, resam-fern spores (Dicranopteris linearis) 34%, sea-teak pollen (Podocarpus polystachyus) 33.8%. Fungal spores Curvularia spp.(26) and Drechslera-like spores (31%). |
| **S./Cenrtal AMERICA** |  |  |  |  |
| **Brazil** |  |  |  |  |
|  | Sao Paolo  Ribeirão Preto | Clinic cases  SPT | 134 children (78 asthma) 4-18 yrs | Dp 84%, Df 81%; Bt 75%. |
|  | Bahia | Clinic cases  SPT | 42 asthma from rural area. Aged 6-35yrs | Dp 43%, Df 38.0% and Bt 43% |
|  | Sao Paolo and Ribeirao Preto | Cross sectional Emergency Department wheezers and controls  Immunocap | 58 asthma children, 2-12 years | Any mite(Dp, Df, Bt) 69%, Bla g 26.3%, cat 5.2%, dog 7%  Sensitization to inhalant allergens risk factor for wheeze OR=2.7 |
|  | Sao Paolo | Cross sectional population based from ISAAC phase 3  SPT | 528 schoolchildren 13 to 14 years old. Asthma=141controls=387 | Ranking from highest rate of sensitization: Dp>Per a, > Bla g,>dog>cat>fungi>grass  Any sensitization a risk factor for asthma (OR 2.81) |
| **Cuba** |  |  |  |  |
|  |  | Cross section, Hospital  SPT, EIA | 148 asthma patients 6-52 years, mean 29 years | D sibony 88%, Dp 87%, A. siro 85%, Bt 85%, Df 83%. |
| **Colombia** |  |  |  |  |
|  | Cartagena | Clinic cases and controls. Elisa, SPT | 175 asthma from lower strata rural areas | Asthma patients: Ascaris lumbricoides tropomyosin 75%, Any mite 83% |
|  | Cartagena | (Risk Factors for Asthma and Allergy in the Tropics)FRAAT general population birth cohort  Immunocap | 290 children from lower economic strata | IgE sensitization at 3 yr old: Bt 33%, Dp19%, Ascaris 26.5%  B. tropicalis-specific IgE and ever wheezing (aOR: 1.47 95% CI: 1.00–2.28, p = 0.05). |
| **Ecuador** |  |  |  |  |
|  | San Lorenzo | Cross sectional schoolchildren rural poor survey.  CAP system | 149 Case and 227 controls  7-19 years old | Cases: Dust mites 22.8%, Cockroach 17.5%, Ascaris 77.2%  Anti-Ascaris IgE associated with wheeze (adj. OR 2.24, p = 0.003) |
|  | Eloy Alfaro and San Lorenzo, Esmeraldas province | Cross sectional population, Rural poor, SPT | 3960 children aged 6 to 16yrs | Wheeze cases: Dp 11%, Per a 14%  Sensitization not associated with wheeze. Predominance of non-atopic wheeze |
| **Venezuela** |  |  |  |  |
|  | Caracas | Cases from Allergy clinic  SPT | 176 all type allergies, 3-77yrs mean 26 | *D. pteronyssinus*t in 97.2%, *B tropicalis* in 91.6%, at least one cockroach extract 83.1%. increased sensitization rate to cockroach allergens in lower socioeconomic levels and those attending the public institution |
| **Puerto Rico** |  |  |  |  |
|  | Puerto Rico | Cross sectional population  SPT | 1496 children <6 to 65 years | All ages asthma: Dp 75%, Df 76%, Bt 70%, E maynei 70%, dog 13%, cat 23%, grass/trees 32% Fungal 18% |
| **Costa Rica** |  |  |  |  |
|  | Costa Rica | Cross sectional, schoolchildren survey ISAAC phase 2, SPT | 208 children 10-13 years | Dp77%, Df62%, cockroach 51%, mixed grass 29%, alternaria 20%  Sensitization to HDM and Alternaria associated with current wheeze |
|  | Costa Rica | Cross sectional, schoolchildren survey, SPT | 439 asthma 6-14yrs | Ascaris 39%, In Ascaris sensitized: HDM 94%, Cockroach 70%  Sensitization to Ascaris associated with at least 1 positive skin test to allergens (odds ratio, 5.15; p < 0.001) |
| **AFRICA** |  |  |  |  |
| **Nigeria** |  |  |  |  |
|  | South west Nigeria | Cross sectional School survey  SPT | 1763 schoolchildren 13-14 yrs old | Positive skin tests to cat hair, cockroach, mango blossom and mouse epithelium associated with asthma than in healthy controls. Overall sensitization in 73% asthma vs 60% controls  No significant differences were observed for HDM allergens |
| **Ethiopia** |  |  |  |  |
|  | Butajira | Population based birth cohort, SPT | 1006 children analyzed at 3 yrs | Overall population: Dp 5.6%, Cockroach 4.2% |
|  | Butajira | Cross sectional population, Butira rural health project, SPT | 7649 >5 yrs | Wheeze weakly associated with allergic sensitization to D. pteronyssinus and cockroach (OR 1.21, 95% CI 0.98–1.51, and 1.27, 95% CI 1.00–1.62, respectively). |
| **Ghana** |  |  |  |  |
|  | Kumasi | Cases and controls from Pediatric clinic, SPT, CAP | 50 children, 9-16 yrs | Df 66%, Dp 62%, cockroach 30%, cat 4%, dog 0%  Dust mite sensitization risk factor for asthma (OR, 10.4; 95% CI, 3.5-30.9). |
|  | Kumasi | Rural and urban school children population  Immunocap | 1848 children 9-16 years. 99 cases of wheeze | Sensitization HDM 51%, Cockroach 59%,. associated with asthma vs controls.  No significance association with sensitization to Ascaris 52.3%, dog 13% and cat 3% (on multiple logistic regression) |
| Abbreviations in alphabetical order: AR=allergic rhinitis, Bla g= Blatella Germanic (German cockroach), Bt= Blomia tropicalis, HK=Hongkong, HDM= house dust mites, Dp = D pteronyssinus, Df=D. farina, OR=Odds Ratio, Per a= Periplaneta americana (American cockroach), SD=standard deviation, SPT=skin prick test. | | | | |
