# Supplementary material for: Particularities of allergy in the Tropics
Source: World Allergy Organ J. 2016 Jun 27;9:20. doi: 10.1186/s40413-016-0110-7 (PMC4924335; doi:10.1186/s40413-016-0110-7)
Supplement: Additional file 4: Table S4. — Food Sensitization in Children Living in the Tropics and Subtropics. (DOCX 30 kb) [file 40413_2016_110_MOESM4_ESM.docx]

| **Additional file 4: Table S4: Food Sensitization in Children Living in the Tropics and Subtropics** | | | | |
| --- | --- | --- | --- | --- |
| Ref/Year | Geographic Location | Study Design/IgE test | Subjects no. and characteristics | Food sensitization data in atopic cases unless otherwise stated |
| **ASIA** |  |  |  |  |
| **HK/China** |  |  |  |  |
| 1  2002 | Hongkong | Hospital clinic. Case Control  Phadia-AutoCAP system | 231 asthma, mean age(SD) 9.3(4.3)yr, 79 age and sex matched controls | Cow milk 20%, egg white 15%, peanut 9%, Soybean 4%, wheat 6%, fish 3%. Clinical food allergy to egg white in 2, and peanut in 2 |
| 2  2007 | Hongkong | Retrospective hospital dermatology clinic.  SPT | 114 eczema 10.2(SD=4.6)yrs | Egg yolk 53%, egg white 42%, Shrimp 35%, Peanuts 31%, crab 29%, lobster 27%, beef/milk/salmon 13%. No mention of clinical food allergy |
| **Taiwan** |  |  |  |  |
| 3  2010 | Taipei | Population based school children  Pharmacia CAP system | 1,500/25,094 positive responses for allergy using ISAAC questionnaire. 7-8year old | Crab 83%, egg white 24.2%, cow milk 22.5%, shrimp 21.4%, wheat 6.7%, buckwheat 4.0%, tuna 2.8%, peanut 2.5%, mussel 2.4%, cod 2.3%, soy 1.6%, Salmon 1.4%. No mention of clinical food allergy |
| 4  2013 | Changhua | Retrospective. Hospital based. h/o of atopy: asthma, AR and eczema  Automated BioIC | 1145 patients: 3 years to adults | 3-6 year age group: soy 26.6%, egg white 23%, crab 12.9%, shrimp 9.4%, Wheat 7.9%. 7-18 year: soy 14.7%, egg white 9.8%, crab 13.2%, shrimp 7.2%, milk 4.9%. >19yrs: soy 11.1%, crab 15.7%, shrimp 4.6%  No mention of clinical food allergy. |
| **Malaysia** |  |  |  |  |
| 5  2004 | Kuala Lumpur | Prospective. Hospital ENT clinic  SPT | 141 children. Asthma, AR, eczema, up to 12 years old  SPT | Prawn 24.6%, crab 24.1%, wheat 22%, squid 10.6%, cockle 9.2%, rice 7.1%, soy 5%, milk 3.5%, egg 3.5%, sardine 2.8%, mutton 2.1%, anchovy 1.4%, banana 1.4%. No mention of clinical food allergy |
| **Singapore** |  |  |  |  |
| 6  2001 | Singapore | Prospective. Hospital clinic  Pharmacia CAP | 75 children. Asthma, AR, eczema, food allergy. Mean age=18.8 (range 1-36) months. | Cow milk 45.9%, egg white 38.7%, soy 4.2%, wheat 12.2%, shrimp 11.5%, peanut 12%. Included cases of clinical food allergy but no details provided |
| 7  2007 | Singapore | Prospective. Hospital clinic  SPT | 227 children. Mean age 5.2(range 0.3-15.4) yrs. Suspected food hypersensitivity + one food SPT positive) | Egg 40%, shellfish 39%, peanuts 13%, Fish 13%, cow milk 12%, sesame seed 9%, wheat 6%, soy 3%. Included cases of food allergy but no details provided |
| 8  2008 | Singapore | Prospective at risk birth cohort. Double blind placebo controlled clinical trial with probiotic intervention  SPT | 253 infants. SPT at 1 year old. | Placebo group (n=121) Egg white 6%, egg yolk 4%, Cow milk 0%, Soy 0%. Intervention group no significant difference |
| **S/Central AMERICA** |  |  |  |  |
| **Brazil** |  |  |  |  |
| 9  2013 | Tocantins | Prospective. Primary health care.  SPT: cow’s milk, casein, beta-lactoglobulin, soybean, egg white, egg yolk, fish, cacao, corn, peanut, shrimp and wheat | 99 children> Mean age 5.34yr (range 1-15) Positive response to ISAAC questionnaire. | Most common food allergen: Cow milk=9.6%. No further details of other allergens |
| 10  2004 | 5 districts of Brazil | Case Control. Allergy clinic  UniCAP Pharmacia | 450 children. 12 to 144 months. Wheezing, food allergy, atopic dermatitis, respiratory allergy. | Fish 29%, Cow milk 23.1%, Wheat 20%, Peanut 14%, Soy 11.8%, Corn 10.6%, Egg 10% |
| **Venezuela** |  |  |  |  |
| 11  1998 | Caracas | Prospective. Hospital based  RAST Pharmacia  Stool microscopic exam for parasites | 125 children. Mean age 6.0 (SD=3.6) years. | The frequency of sensitization to allergens not reported. Levels of cow’s milk and egg specific IgE significantly higher in presence Giardia lamblia infections. Dust mite sensitization was similar between groups. |
| 12  2014 | Delta Amacuro State | Prospective. Cross sectional preschool  SPT | 169 children. 3 to 6 years. 39 children with atopic dermatitis. | Cow milk 76.9%, hen egg 48.7%, |
| **AFRICA** |  |  |  |  |
| **Ghana** |  |  |  |  |
| 13  2011 | Greater Accra Region | Cross sectional. Case Control. Schoolchildren  ISAAC II  SPT, ImmunoCAP Phadia when SPT positive  Stool examination | 1431 children 5-16 years. Urban and rural schools | Whole cohort: 5% at least one SPT positive. Peanut 2%, Pineapple 2%, Pawpaw 1%, Orange 1%, Mango 0.8%, Banana 0.4%, Apple 0.3%. No clear association with symptoms of food allergy. Association between SPT and sIgE in urban but not rural subjects. Differences unrelated to helminths. |
| 14. | Soutthern Ghana | Cross sectiona. School children | 1604 schoolchildren 5-16 years old  SPT  ImmunoCAP  ImmunoCAP Peanut components | Peanut: SPT: 2%, Immunocap 17.5%  Subset of 43: Ara h 1, 2, 3, 9 specific IgE levels low, <1.3kU/L. Six with moderately strong Ara h 9 levels |
| Abbreviations in alphabetical order: AR=allergic rhinitis, BioIC=automated microfluidic-based immunoassay system, ENT=Ear Nose Throat, SD=standard deviation, SPT=skin prick test | | | | |

1. Leung TF[[1](#_ENREF_1)], Lam CW, Chan IH, Li AM, Tang NL. Sensitization to common food allergens is a risk factor for asthma in young Chinese children in Hong Kong. J Asthma. 2002 Sep;39(6):523-9.
2. Hon KL, Leung TF, Lam MC, Wong KY, Chow CM, Ko WS, Fok TF, Leung AK. Eczema exacerbation and food atopy beyond infancy: how should we advise Chinese parents about dietary history, eczema severity, and skin prick testing? Adv Ther. 2007 Mar-Apr;24(2):223-30.
3. Wan KS, Yang W, Wu WF. A survey of serum specific-lgE to common allergens in primary school children of Taipei City. Asian Pac J Allergy Immunol. 2010 Mar;28(1):1-6
4. Yong SB, Wu CC, Tzeng YC, Hung WC, Yang KD. Different profiles of allergen sensitization in different ages and geographic areas in Changhua, Taiwan. J Microbiol Immunol Infect. 2013 Aug;46(4):295-301.
5. Gendeh BS, Mujahid SH, Murad S, Rizal M. Atopic sensitization of children with rhinitis in Malaysia. Med J Malaysia. 2004 Oct;59(4):522-9.
6. Khoo J, Shek LP, Khor ES, Wang DY, Lee BW. Pattern of sensitization to common environmental allergens amongst atopic Singapore children in the first 3 years of life. Asian Pac J Allergy Immunol. 2001 Dec;19(4):225-9.
7. Chiang WC, Kidon MI, Liew WK, Goh A, Tang JP, Chay OM. The changing face of food hypersensitivity in an Asian community. Clin Exp Allergy. 2007 Jul;37(7):1055-61.
8. Soh SE, Aw M, Gerez I, Chong YS, Rauff M, Ng YP, Wong HB, Pai N, Lee BW, Shek LP. Probiotic supplementation in the first 6 months of life in at risk Asian infants--effects on eczema and atopic sensitization at the age of 1 year. Clin Exp Allergy. 2009 Apr;39(4):571-8.
9. Baldaçara RP, Fernandes Mde F, Baldaçara L, Aun WT, Mello JF, Pires MC. Prevalence of allergen sensitization, most important allergens and factors associated with atopy in children. Sao Paulo Med J. 2013;131(5):301-8.
10. Naspitz CK, Solé D, Jacob CA, Sarinho E, Soares FJ, Dantas V, Mallozi MC, Wandalsen NF, Borges W, Rocha Filho W; Grupo PROAL. [Sensitization to inhalant and food allergens in Brazilian atopic children by in vitro total and specific IgE assay. Allergy Project--PROAL]. J Pediatr (Rio J). 2004 May-Jun;80(3):203-10.
11. Di Prisco MC, Hagel I, Lynch NR, Jiménez JC, Rojas R, Gil M, Mata E. Association between giardiasis and allergy. Ann Allergy Asthma Immunol. 1998 Sep;81(3):261-5.
12. Hagel I, Puccio F, López E, Lugo D, Cabrera M, Di Prisco MC. Intestinal parasitic infections and atopic dermatitis among Venezuelan Warao Amerindian pre-school children. Pediatr Allergy Immunol. 2014 May;25(3):276-82.
13. Obeng BB, Amoah AS, Larbi IA, Yazdanbakhsh M, van Ree R, Boakye DA, Hartgers FC. Food allergy in Ghanaian schoolchildren: data on sensitization and reported food allergy. Int Arch Allergy Immunol. 2011;155(1):63-73.
14. Amoah AS, Obeng BB, Larbi IA, Versteeg SA, Aryeetey Y, Akkerdaas JH, Zuidmeer L, Lidholm J, Fernández-Rivas M, Hartgers FC, Boakye DA, van Ree R, Yazdanbakhsh M. Peanut-specific IgE antibodies in asymptomatic Ghanaian children possibly caused by carbohydrate determinant cross-reactivity. J Allergy Clin Immunol. 2013 Sep;132(3):639-47.
