# Supplementary material for: Particularities of allergy in the Tropics
Source: World Allergy Organ J. 2016 Jun 27;9:20. doi: 10.1186/s40413-016-0110-7 (PMC4924335; doi:10.1186/s40413-016-0110-7)
Supplement: Additional file 5: Table S5. — Overview of tropical studies examining pet sensitization in children. (DOCX 24 kb) [file 40413_2016_110_MOESM5_ESM.docx]

| **Additional file 5: Table S5: Overview of tropical studies examining pet sensitization in children** | | | | | | | |
| --- | --- | --- | --- | --- | --- | --- | --- |
| Year | First Author ^(Ref)^ | Geographic Location | Study Design | Characteristics of study population | Number of subjects | Pet sensitization, prevalence (%) | Other significant findings |
| 2013 | Baldacara^12^ | Palmas, Brazil | Cross-sectional | Age 1-15 years  Seen at paediatric outpatient clinics for any reason | 94 | Cats 28.7%  Dogs 21.3% | 57% of patients with positive food/inhalant SPT had pet exposure.  Exact relationship between pet exposure and positive SPT to pets was unclear.  Der p sensitization 34% |
| 2013 | Yuenyongviwat^13^ | Thailand | Cross-sectional | Mean age 7.54 years in year 2004, 7.73 years in year 2009  Children with asthma, seen at the paediatric allergy clinic of university hospital | 99 in year 2004, 99 in year 2009 | Cat 10.1% (year 2004) -> 7.1% (year 2009)  Dog 0% | Der p sensitization 50.5% (year 2004) -> 48.5% (year 2009)  Der f sensitization 52.5% (year 2004) -> 48.5% (year 2009) |
| 2013 | Oluwole^14^ | Nigeria | Cross-sectional in first phase, case-control in second phase | Children aged 13-14 years  Recruited from high schools in urban and rural communities | 1736 in first phase  85 cases of asthma and 85 healthy controls in second phase | - | Cat in home in the past year associated with asthma (aOR 1.56, p=0.03)  Asthmatics were more likely to be sensitized to cat (OR 2.00) |
| 2012 | Chiang^15^ | Singapore | Cross-sectional | Age up to 18 years, mean 7.8 years  Children with chronic rhinitis  Seen at outpatient specialist clinics of Allergy, Respiratory  or Otolaryngology departments of tertiary hospital  Tested to dog dander only if above 4 years of age | 6660 | Cats 6.4% of 5012 children with allergic rhinitis  Dogs 2.2% of 5012 children with allergic rhinitis | 75.9% of patients (n=5012) had allergic rhinitis  Mean age of sensitization to cat/dog dander was 8.9 years  House dust mite sensitization 90.8% |
| 2012 | Morfin-Maciel^16^ | Mexico City, Mexico | Cross-sectional | Age 3-18 years, mean 11.5 years  Children with asthma.  First attendance to Allergy Department | 260 |  | Cat sensitization associated with more severe asthma  Dust mite sensitization was the most prevalent |
| 2012 | Rigalt^17^ | Guatemala | Cross-sectional | Age 5-15 years, mean 8.3years  Children with allergic rhinitis and/or asthma | 461 | Cat 14%  Dog 8% | Der p sensitization 44%  Der f sensitization 43% |
| 2011 | Leung^18^ | Hong Kong | Cross-sectional | Age 5-18 years, mean 12.4 years  Children with asthma  Seen at paediatric allergy clinic of university teaching hospital | 159 | Cat 28.7% | Keeping of cat or dog strongly associated with presence of Fel d 1 in house dust samples  Der p sensitization 78.3% |
| 2011 | Hernandez-Venegas^19^ | Mexico | Cross-sectional | Age 7-18 years, mean 10.1 years  Children with asthma  First attendance to Allergy Department | 104 | Cat 17.2%  Dog 5.3% | Asthma exacerbated by animal exposure in 16.3% of patients  Der p sensitization 94.6%  Der f sensitization 89.2% |
| 2011 | Stevens^20^ | Ghana | Case-control | Age 9-16 years  Children with asthma or current wheeze, from schools  sIgE using ImmunoCAP done for sensitization | Subjects who could provide serum for specific IgE: 88 cases, 93 controls | Cat 3.4% in cases, 1.1% in controls (p>0.05)  Dog 14.8% in cases, 7.5% in controls (p>0.05) | No significant difference in presence of cat/dog sIgE between urban affluent, urban poor and rural/suburban subjects (numbers very small)  Dust mite 51.1% in cases, 16.1% in controls |
| 2004 | Naspitz^21^ | Brazil | Case-control | Age 1-12 years  RAST tests done for cat/dog sensitization | 457 atopic children; 62 healthy controls (negative SPT and no atopic disease) | Cat 12.2% in atopic children, 8.1% in controls  Dog 8.1% in atopic controls, 3.2% in controls | Der p sensitization 67.8%  Der f sensitization 66.5%  Blo t sensitization 57.1% |
| 2004 | Sritipsukho^22^ | Thailand | Cross-sectional | Age 23 months to 15 years (mean 8.1 years)  Children with asthma  and/or allergic rhinitis  Attending Allergy Clinic in university hospital | 120 | Cat 13%  Dog 8% | Der p 79%, Der f 69% |
| 2002 | Wong^23^ | Hong Kong, Guangzhou | Cross-sectional | Age 9-11 years  Children from schools in community | 1341 in Hong Kong  1094 in Guangzhou | Cat 3.7% in Hong Kong, 4.3% in Guangzhou | Cat sensitization is a risk factor for current wheeze in Guangzhou, but not Hong Kong  Study compared findings to Beijing, a temperate city in China. Cat sensitization was highest in Beijing and was associated with bronchial hyperresponsiveness  Der p and Der f sensitization were far more common than cat in Hong Kong and Guangzhou, and only slightly more common in Beijing |
| 2002 | Soto-Quiros^24^ | Costa Rica | Cross-sectional | Age 10-13 years    Children from schools in community | 208 |  | Positive SPT to cat associated with allergic rhinitis  Positive SPT to dog associated with eczema  Strongest predictor for current wheeze, eczema and allergic rhinitis was positive SPT to Der p |
| 2002 | Leung^25^ | Hong Kong | Case-control | Age <15 years (mean 9.8 years)  Children with physician-diagnosed asthma, managed in university teaching hospital | 170 with asthma, 57 healthy controls | Cat 40%  Dog 22% | Positive SPT to cat associated with asthma (p=0.03)  Der p sensitization 88%  Der f sensitization 86% |
| 2001 | Khoo^26^ | Singapore | Cross-sectional | Age below 3 years  Children with atopy  Seen at paediatric clinic of tertiary hospital | 75 | Cat 1.5% (3.6% in infants, 0% in age 1-3 years)  Dog 6.5% (7.2% in infants, 4.3% in age 1-3 years) | Der p sensitization 31.4%  Blo t sensitization 25.5% |
| 2001 | Addo-Yobo^27^ | Ghana | Case-control | Age 8-17 years  Cases recruited from paediatric asthma clinic in tertiary referral centre  Controls recruited from local school | 50 with asthma, 50 healthy controls | Cat 4% in asthmatics, 2% in controls  Dog 0% in asthmatics, 2% in controls | No significant association between dog or cat sensitization, and asthma  Cat ownership in 42% of asthmatics and 30% of controls; dog ownership in 24% of asthmatics and 46% of controls. However allergen levels in homes were low (pets in Ghana kept outdoors)  Most children with negative SPT to cat and positive CAP to cat, did not have Fel d1 on ELISA  Sensitization to dust mites was the strongest predictor for asthma |
| 1998 | Soto-Quiros^28^ | Costa Rica | Case-control | Age unclear  Cases and controls recruited from schools  Sensitization dependent on result of MAST and/or RAST tests | Cases had asthma; controls were not asthmatic  171 tested to MAST, 347 tested to RAST |  | No difference between cases and controls when considering cat or dog sensitization, using either method  Sensitization to Der f was the most prevalent, approximately 50% |
| 1997 | Chew^29^ | Singapore | Cross-sectional | Age 3-5 years, and 6-14 years  Children with asthma  Seen at paediatric asthma outpatient clinic of tertiary hospital | 58 children aged 3-5 years  118 children aged 6-14 years | Cat 27.6% in children aged 3-5 years, 25.4% in children aged 6-14 years  Dog 17.2% in children aged 3-5 years, 33.9% in children aged 6-14 years | Dust mite sensitization exceeded cat/dog sensitization for all age groups and all dust mite varieties |
